# The role of localised prostate cancer treatment in renal transplant patients: A systematic review

**DOI:** 10.1002/bco2.276

**Published:** 2023-08-09

**Authors:** Anthony Dat, Gavin Wei, Simon Knight, Weranja Ranasinghe

**Affiliations:** ^1^ Department of Urology Monash Health Melbourne Australia; ^2^ Department of Transplantation, Centre for Evidence in Transplantation John Radcliffe Hospital Oxford UK

**Keywords:** kidney, prostate cancer, renal, transplantation

## Abstract

**Objective:**

To systematically review and critically appraise all treatment options for localised prostate cancer in renal transplant candidates and recipients.

**Method:**

A systematic review was conducted adhering to PRISMA guidelines. Searches were performed in the Cochrane Library, Embase, Medline, the Transplant Library and Trip database for studies published up to September 2022. Risk of bias was assessed with the Cochrane Risk of Bias in Non‐Randomised Studies of Interventions for non‐randomised studies tool.

**Results:**

A total of 60 studies were identified describing 525 patients. The majority of studies were either retrospective non‐randomised comparative or case series/reports of poor quality. The vast majority of studies were focussed on prostate cancer after renal transplantation. Overall, 410 (78%) patients underwent surgery, 93 (18%) patients underwent radiation therapy or brachytherapy, one patient underwent focal therapy (high‐intensity frequency ultrasound) and 21 patients were placed on active surveillance. The mean age was 61 years old, the mean PSA level at diagnosis was 9.6 ng/mL and the mean follow‐up time was 31 months. The majority of patients had low‐risk disease with 261 patients having Gleason 6 prostate cancer (50%), followed by 220 Gleason 7 patients (42%). All prostate cancer mortality cases were in high‐risk prostate cancer (≥Gleason 8). The cancer‐specific survival results were similar between surgery and radiotherapy at 1 and 3 years.

**Conclusion:**

Localised prostate cancer treatment in renal transplant patients should be risk stratified. Surgery and radiation treatment for localised prostate cancer in renal transplant patients appear equally efficacious. Given the limitations of this study, future research should concentrate on developing a multicentre RCT with long‐term registry follow‐up.

## 1 INTRODUCTION

Prostate cancer has an increasing incidence in renal transplant recipients (RTRs) because of a variety of factors including pre‐transplant screening, increasing age of recipients and prolonged survival after transplantation. The incidence of kidney transplants has increased with the highest surge in those aged 45 to 65 years old, corresponding to the index group for prostate cancer screening.^1^ Although it is undeniable that renal transplantation has improved life expectancy in chronic renal disease patients, the management of prostate cancer in this group is controversial. Multiple unknowns include the impact of immunosuppression, the role of cancer screening and the timing and type of prostate cancer treatment. Recent studies suggest that prostate cancer outcomes are no worse in RTR compared with their non‐transplant counterparts.^2^, ^3^ The aim of this study was to review and critically appraise all treatment options for localised prostate cancer in renal transplant candidates and recipients.

## 2 METHODS

### 2.1 Study design

The study was a systematic review reported according to PRISMA 2020 standards.^4^ A study protocol was registered with PROSPERO (CRD42022311393).

### 2.2 Eligibility criteria

Eligible participants included adult patients with a diagnosis of localised prostate cancer pre‐ or post‐renal transplantation. Patients with metastatic disease at time of diagnosis were excluded. Interventions included active surveillance, surgery, radiation ± androgen deprivation therapy and focal therapies. All prospective and retrospective comparative and non‐comparative studies were included.

### 2.3 Types of outcome measures

#### 2.3.1 Primary outcomes

- Prostate cancer‐specific survival
- Overall survival

#### 2.3.2 Secondary outcomes

- Graft outcomes: graft renal function, graft survival and complications
- Biochemical recurrence rate
- Positive margin rate
- Prostate cancer treatment complications including urinary incontinence, erectile dysfunction, radiation cystitis and proctitis

### 2.4 Information sources

The following databases were searched:

- OVID MEDLINE (1946 onwards)
- OVID EMBASE (1974 onwards)
- Trip Database
- Cochrane Library
- The Transplant Library

### 2.5 Search strategy

OVID MEDLINE and OVID EMBASE were searched using the search string found in Table A1. This strategy was adapted to search the Cochrane Library, the Transplant Library and the Trip database. Full articles with relevant clinical information were retrieved and reviewed. The bibliographies of all retrieved and relevant publications identified by the above strategies were searched for further studies. The above strategy was also used to search abstract proceedings for major urological conferences including EAU, AUA, BAUS and USANZ. There was no language restriction.

### 2.6 Study records

#### 2.6.1 Selection process

Two reviewers (AD and GW) searched the above information sources independently and assessed identified studies for inclusion. The full study text was reviewed when it could not be clearly excluded on the basis of its title and abstract. A study was included when both reviewers independently assess it as satisfying the eligibility criteria from the full text. A third reviewer (WR) mediated in the event of a disagreement following discussion.

#### 2.6.2 Data management

Data extraction was processed onto a data extraction form. Duplicate studies were only included once.

#### 2.6.3 Data collection process

Authors independently extracted data on the trial inclusion criteria using standardised forms. The following data were extracted: author, year of publication, country, study period, inclusion criteria, total number of people and cancer treatment modality.

#### 2.6.4 Data items

Data extracted included sample size, baseline patient characteristics including age, baseline immunosuppression, PSA level, cancer grade and stage. Surgical data included approach, estimated blood loss, length of stay and presence of pelvic lymph node dissection. Radiation data included total dose and presence of neoadjuvant ADT.

### 2.7 Outcomes and prioritisation

#### 2.7.1 Primary outcomes

- The primary outcome was survival (prostate cancer‐specific survival and overall survival). Cancer‐specific survival was defined as deaths identified as being due to prostate cancer. Overall survival was defined as death due to any cause. The time points for survival were at 1 and 3 years.

#### 2.7.2 Secondary outcomes

- Prostate cancer treatment complications were collated according to the Clavien Dindo Classification.^5^ Short‐term complications include organ injury and bleeding. Long‐term complications include urinary incontinence, urethral stricture, erectile dysfunction, radiation cystitis and proctitis.
- Renal graft complications were collated according to the Clavien Dindo Classification for surgical complications and Common Terminology Criteria for Adverse Events (CTCAE) for radiation complications.^6^
- Biochemical recurrence rate and positive margin rate

### 2.8 Risk of bias in individual studies

Risk of bias for each included trial was assessed by the same initial reviewers. Risk of bias was assessed using the Cochrane Risk of Bias in Non‐Randomised Studies of Interventions (ROBINS‐1) for non‐randomised studies.^7^ Case series were assessed using the Canada Institute of Health Economics Quality Appraisal Tool for case series.^8^

### 2.9 Data synthesis

Data summary was provided with tables and graphs. A narrative synthesis explored the relationship and findings of the included studies.

### 2.10 Pooled treatment effects

#### 2.10.1 Dichotomous and continuous outcomes

Dichotomous data (e.g. survival at 1 and 3 years) were pooled with a single‐arm meta‐analysis with weighting according to inverse variance using a random effects model. Key continuous outcomes (e.g. PSA) were analysed using mean. Considering there was a lack of similar comparative studies, a double‐arm meta‐analysis was not performed.

### 2.11 Dealing with missing data

Attempts were made to make contact with individual study authors when missing data were identified.

### 2.12 Assessment of heterogeneity

The extent and impact of between‐study heterogeneity were assessed by inspecting the forest plots and by calculating the tau‐squared and I‐squared statistics respectively. The 95% confidence intervals around tau‐squared and I‐squared were calculated to judge our confidence about these metrics. We adopted the following I‐squared thresholds to assess heterogeneity^9^:

- 0 to 40%: heterogeneity may not be important
- 30–60%: may represent mild heterogeneity
- 50–90%: may represent moderate heterogeneity
- 75–100%: considerate heterogeneity

All analyses were run in R Studio Metafor Package Version 4.2.1 (R Foundation for Statistical Computing, Vienna, Austria).

A subgroup analysis was performed according to treatment type. A sensitivity analysis examining quality components and risk of bias was not possible because of the lack of randomised controlled trials.

### 2.13 Meta‐bias (es)

Considering the lack of RCTs, it was not possible to perform a funnel plot. Selection and publication bias was discussed in a narrative fashion as part of the critical appraisal process.

### 2.14 Confidence in cumulative evidence

Strength of body of evidence was assessed using the Grading of Recommendations, Assessment, Development and Evaluation (GRADE).^10^ Each rating addresses key elements including the overall quality of evidence, magnitude of the effect, certainty of the results, the impact of patient values and preferences and certainty of these values and preferences.

## 3 RESULTS

### 3.1 Search selection

**FIGURE 1:**
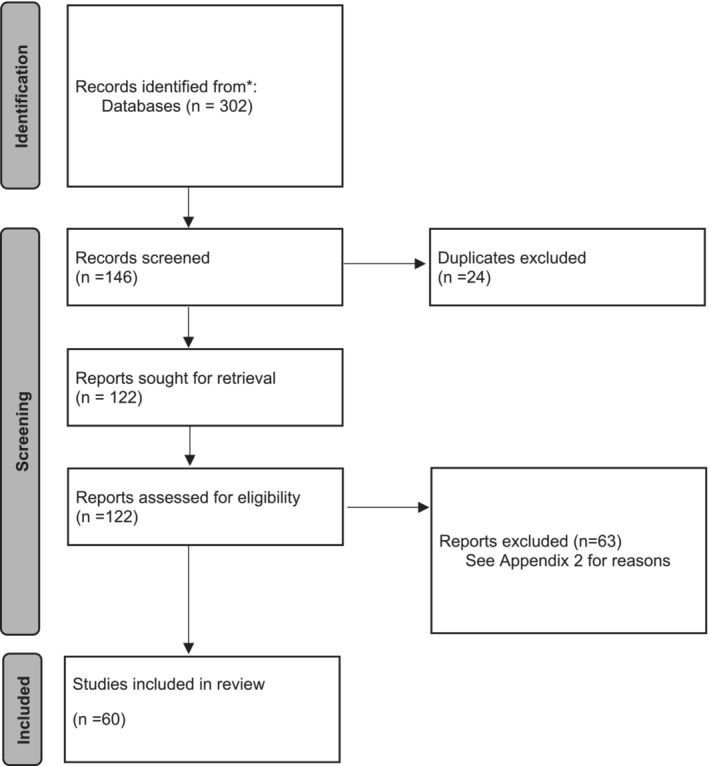
Search selection diagram.

Figure 1 details the search selection process. The search of listed databases identified 302 studies using a combination of search terms ‘renal transplant’ AND ‘prostate cancer’ or their medical subject heading (MeSH) equivalent. This was then combined with the various search terms for prostate cancer interventions and controls to lead to 146 abstracts eligible for screening. Of these, 24 duplicates were excluded. 122 reports were assessed for eligibility with 63 excluded. Table A2 lists the reasons for exclusion. A total of 60 studies were included in the systematic review.

### 3.2 Study characteristics and results of individual studies

The 60 studies included a total of 525 patients. Table 1 lists all included studies. All studies were either retrospective non‐randomised comparative or case series/reports. Four studies were published in the 1990s, the remaining were published in the 21st Century.^11^, ^12^, ^28^, ^37^ Studies recruited patients between 1980 and 2021. The vast majority of studies were focussed on prostate cancer after renal transplantation. PSA screening for renal transplant patients prior to prostate cancer diagnosis was mentioned in only seven studies with the most common protocol being yearly PSA and digital rectal examination from the age of 50.^12^, ^16^, ^26^, ^63^, ^64^, ^67^, ^68^

**TABLE 1:** Included studies.

Study	Year	Study country	Timing of prostate cancer treatment	IDEAL classification	Treatment approach	Study type	Level of evidence	Accrual years	Patients
Kinahan^11^	1991	Canada	Post‐transplant	2A	Open	Case Series	4	1987–1989	3
Morton^12^	1995	USA	Post‐transplant	2A	Open	Case Series	4	1990–1993	2
Campagnari^13^	2002	Brazil	Post‐transplant	2A	Open	Case Series	4	1992–2002	2
Hafron^14^	2004	USA	Post‐transplant	2A	Perineal	Case Series	4	1999–2004	7
Kleinclauss^15^	2008	France	Post‐transplant	2B	Open	Retrospective comparative	3	1996–2007	20
Antonopoulos^16^	2008	Brazil	Post‐transplant	2A	Open	Case Series	4	2004–2007	8
Robert^17^	2009	France	Post‐transplant	2A	Lap	Retrospective comparative	3	2007–2008	9
Maestro^18^	2009	Spain	Post‐transplant	2A	Lap	Case Series	4	2006–2007	2
Hoda^19^	2010	Germany	Post‐transplant	2A	Open	Retrospective comparative	3	2001–2007	16
Smith^20^	2011	USA	Post‐transplant	2A	Robotic	Case Series	4	2005–2008	3
Polcari^21^	2012	USA	Post‐transplant	2B	Robotic	Case Series	4	2004–2010	7
Heidenreich^22^	2014	Germany	Post‐transplant	2A	Open and Perineal	Retrospective comparative	3	2000–2011	23
Le Clerc^23^	2015	France	Post‐transplant	2A	Robotic	Case Series	4	2009–2013	12
Iizuka^24^	2016	Japan	Post‐transplant	2A	Robotic	Case Series	4	2011–2015	3
Moreno Sierra^25^	2016	Spain	Post‐transplant	2A	Robotic	Case Series	4	2012–2013	4
Pettenati^26^	2016	France	Post‐transplant	2A	Open, Robotic, LapEBRT and BrachytherapyAS	Retrospective comparative	3	2000–2013	24
Beyer^27^	2016	Germany	Post‐transplant	2A	RRP	Case Series	4	1992–2013	20
Yiou^28^	1999	France	Post‐transplant	1	Perineal	Case Report	4	1997	1
Shah^29^	2006	USA	Post‐transplant	1	LRP	Case Report	4	NA	1
Jhaveri^30^	2008	USA	Post‐transplant	1	Robotic	Case Report	4	2008	1
Doerfler^31^	2009	France	Post‐transplant	1	LRP	Case Report	4	2006	1
Wagener^32^	2012	Germany	Post‐transplant	1	Robotic	Case Report	4	2010	1
Saema^33^	2010	Thailand	Post‐transplant	1	LRP	Case Report	4	2010	1
Jenjitranant^34^	2014	Thailand	Post‐transplant	1	Robotic	Case Report	4	2014	1
Ghazi^35^	2012	USA	Post‐transplant	1	Robotic	Case Report	4	2012	1
Detti^36^	2011	Italy	Post‐transplant	1	Open	Case Report	4	2011	1
Multanen^37^	1998	Finland	Post‐transplant	1	Open	Case Report	4	1998	1
Zeng^38^	2018	USA	Post‐transplant	1	Robotic	Case Report	4	2018	1
Plagakis^39^	2016	Australia	Post‐transplant	1	Robotic	Case Report	4	2016	1
Minami^40^	2020	Japan	Post‐transplant	1	Robotic	Case Report	4	2020	1
Tugcu^41^	2017	Turkey	Post‐transplant	1	Robotic	Case Report	4	2018	1
Thomas^42^	2007	USA	Post‐transplant	2A	LRP	Case Series	4	1999–2006	3
Sirisopana^43^	2021	Thailand	Post‐transplant	2A	Open, Robotic, Lap	Case Series	4	2008–2020	5
Mistretta^44^	2019	Italy	Post‐transplant	2A	Robotic	Case Series	4	2012–2016	9
Shahait^45^	2021	USA	Post‐transplant	2A	Robotic	Case Series	4	2014–2019	14
Kobari^46^	2021	Japan	Post‐transplant	1	Robotic	Case Report	4	2021	1
Leonard^47^	2020	France	Post‐transplant	2B	Robotic	Retrospective comparative	3	2008–2016	27
Iwamoto^48^	2018	Japan	Post‐transplant	2A	Open, Robotic, Lap	Case Series	3	2008–2017	13
Fang^49^	2018	China	Post‐transplant	1	Robotic	Case Report	4	2016	1
Felber^50^	2020	France	Post‐transplant	2B	Robotic	Retrospective comparative	3	2008–2017	39
Aboumohamed^51^	2015	USA	Post‐transplant	2A	Robotic	Case Series	4	2015	5
Wang^52^	2017	Singapore	Post‐transplant	1	Robotic	Case Report	4	2017	1
Marra^53^	2022	UK, Italy, France, Germany, Turkey	Post‐transplant	2B	Robotic	Case Series	4	2009–2019	41
Binsaleh^54^	2011	Saudi Arabia	Post‐transplant	2B	EBRT	Case Series	4	1980–2006	8
Beydoun^55^	2014	Australia	Post‐transplant	2A	Brachytherapy	Case Series	4	2002–2012	4
Iizuka^56^	2016	Japan	Post‐transplant	2A	EBRT	Case Report	4	2012	2
Iizuka^57^	2016	Japan	Post‐transplant	2A	Brachytherapy	Case Report	4	2012	2
Rosenfelder^58^	2014	UK	Post‐transplant	1	EBRT	Case Report	4	2014	1
Mouzin^59^	2004	France	Post‐transplant	1	EBRT	Case Series	4	1999–2003	8
Detti^60^	2022	France	Post‐transplant	2A	EBRT	Case Series	4	1998–2017	6
Ileana^61^	2020	Mexico	Post‐transplant	2A	EBRT	Case Series	4	2013–2018	2
Tasaki^62^	2019	Japan	Post‐transplant	2A	Brachytherapy	Case Series	4	2007–2018	3
Rivero‐Belenchon^63^	2018	Spain	Post‐transplant	2A	Brachytherapy	Case Series	4	2000–2015	8
Gojdic^64^	2019	Slovakia	Post‐transplant	2A	EBRT Brachytherapy	Case Series	4	2003–2016	4
Lledo^65^	2005	Spain	Post‐transplant	1	HIFU	Case Report	4	2005	1
Kocak^66^	2009	Turkey	Pre‐transplant	1	LRP	Case Report	4	2009	1
Chahwan^67^	2017	France	Pre‐transplant	2B	Open, Perineal, Lap EBRT Brachytherapy	Case Series	4	2003–2015	52
Tillou^68^	2014	France	Pre‐transplant	2A	Open, Lap	Case Series	4	2003–2013	19
Harada^69^	2017	Japan	Pre‐transplant	1	Brachytherapy	Case Report	4	2014	1
Bratt^3^	2020	Sweden	Post‐transplant	4	Open, RARP, Brachy, EBRTx	Retrospective comparative	1	1992–2017	65

Overall, 410 (78%) patients underwent surgery, 93 (18%) patients underwent radiation therapy or brachytherapy, one patient underwent focal therapy (HIFU) and 21 patients were placed on active surveillance. The mean age was 61 years old, the mean PSA level at diagnosis was 9.6 ng/mL and the mean follow‐up time was 31 months. The majority of patients had low‐risk disease with 261 patients having Gleason 6 prostate cancer (50%), followed by 220 Gleason 7 patients (42%). All prostate cancer mortality cases were in high‐risk prostate cancer (≥Gleason 8). Thirty‐three (8%) patients had high‐risk prostate cancer (Gleason 8 or higher). The vast majority of patients (*n* = 415 patients, 80%) had pathologically or clinically organ‐confined localised disease (pT2 or cT2 or less). This was followed by 85 patients (16%) with extraprostatic extension or seminal vesicle invasion (pT3).

### 3.3 Quality assessment

The quality of the included studies was generally low. Eight retrospective comparative papers had level of evidence III with control groups, and the remaining 52 case series and case report studies had level IV evidence. Using the ROBINS‐1 tool, all studies were classified as critical risk of bias.^7^ Table A3 summarises the risk of bias quality in the retrospective comparative studies. Considering the retrospective nature of these trials, there were global concerns with serious and critical risk of bias with confounding, patient selection and outcome measurement. All studies did not have an objective measure of functional outcomes such as continence or erectile function. The case report and case series were assessed using the Canada Institute of Health Economics Quality Appraisal Tool.^8^ The quality of the studies was generally poor. Table A4 summarises the risk of bias quality assessment.

### 3.4 Results of syntheses

The outcome results were broken down by treatment group and timing of prostate cancer treatment (pre‐transplant vs. post‐transplant).

### 3.5 Surgery post‐transplant

Table 2 summarises all surgical patients with further supplementary data in Table A5. There were 43 post‐transplant surgical studies with a total of 330 patients. The mean PSA was 9 ng/mL with a patient mean age of 62 years. The mean time from renal transplant to prostate cancer was 117 months. The mean follow‐up period was 29 months. The most common type of prostate cancer surgery was robotic (60%) followed by open (29%). The vast majority of prostate cancer (74%) was organ confined. From a grading perspective, 48% of patients had Gleason 7 disease followed by 42% having Gleason 6 disease. PLND was performed in one quarter of prostate cancer surgeries with the majority of these being the contralateral pelvic lymph node packet only. The reasons for pelvic lymph node dissection were poorly recorded.

**TABLE 2:** Included surgical studies.

Study	Year	Study country	Surgical approach	Study type	Level of evidence	Accrual years	Patients	Mean PSA (range), ng/mL	Mean age (range), years	Mean follow‐up (range), months
Pre‐transplant										
Kocak	2009	Turkey	Lap	Case Report	4	2009	1	3.2	57	3
Tillou	2014	France	Open/Lap	Case Series	4	2003–2013	19	8.5 (4.8–20)	61.7 (51.4–71.1)	38 (6–77.9)
			Open				14	8.09 (4.8–20)	62.6 (51.7–71.1)	Not recorded
			Lap				5	9.07 (4.97–14)	59.2 (51.4–68.6)	Not recorded
Chahwan	2017	France	Open/Lap/Perineal	Case Series	4	2003–2015	46	7^a^ (3.6–25)	59.8^a^ (45.6–72.9)	Not recorded
			Open				28	7.2^a^	58^a^	27^a^ (4.1–90)
			LRP				15	6.9^a^	60^a^	37^a^ (7.7–72)
			Perineal				3	14.13 (5.9–25)	61.5 (49–69.9)	35.8 (23.5–45.9)
Post‐transplant										
Kinahan	1991	Canada	Open	Case Series	4	1968–1999	3	Not recorded	60 (56–64)	10.6 (3–24)
Morton	1995	USA	Open	Case Series	4	1974–1992	2	12.7 (9.4–16)	64 (63–65)	27 (24–30)
Multanen	1998	Finland	Open	Case Report	4	1998	1	13	51	18
Yiou	1999	France	Perineal	Case Report	4	1997	1	5	56	10
Campagnari	2002	Brazil	Open	Case Series	4	1992–2002	2	7.25 (4.4–10.1)	66 (57–75)	16.5 (9–24)
Hafron	2005	USA	Perineal	Case Series	4	1999–2004	7	7.9 (5.6–10)	62.3 (55–74)	22 (2–130)
Shah	2006	USA	LRP	Case Report	4	NA	1	5.7	50	36
Thomas	2007	USA	LRP	Case Series	4	1999–2006	3	11.05	58.3 (48–64)	16.6 (14–18)
Antonopoulos	2008	Brazil	Open	Case Series	4	2004–2007	8	4.5 (1.6–7)	59.6 (49–67)	11.9 (2–30)
Jhaveri	2008	USA	Robotic	Case Report	4	2008	1	8.5	54	1.5
Kleinclauss	2008	France	Open	Retrospective comparative	3	1996–2007	20	7.1 (4.5–9.5)	60.4	29
Doerfler	2009	France	LRP	Case Report	4	2006	1	7.1	63	18
Maestro	2009	Spain	Lap	Case Series	4	2006–2007	2	6.95 (4.2–9.7)	59.5 (56–63)	30 (24–36)
Robert	2009	France	Lap	Retrospective comparative	3	2007–2008	9	8.6 (2.6–26)	61.44 (54–67)	11.9
Hoda	2010	Germany	Open	Retrospective comparative	3	2001–2007	16	4.7	61.8 (51–66)	25.2
Saema	2010	Thailand	LRP	Case Report	4	2010	1	10.8	64	12
Detti	2011	Italy	Open	Case Report	4	2011	1	6	50	Not recorded
Smith	2011	USA	Robotic	Case Series	4	2005–2008	3	Not recorded	54.3 (48–61)	13
Ghazi	2012	USA	Robotic	Case Report	4	2012	1	6.93	68	
Polcari	2012	USA	Robotic	Case Series	4	2004–2010	7	6.2 (3.5–12.8)	63.3 (55–72)	16 (12–29)
Wagener	2012	Germany	Robotic	Case Report	4	2010	1	12.4	71	9
Heidenreich	2014	Germany	Open	Retrospective comparative	3	2000–2011	16	4.5 (3–17.5)	64 (59–67)	48 (45–141)
Heidenreich	2014	Germany	Perineal	Retrospective comparative	3	2000–2011	7	4.3 (3.6–10.5)	64 (52–69)	39 (10–85)
Jenjitranant	2014	Thailand	Robotic	Case Report	4	2014	1	11.5	73	1
Aboumohamed	2015	USA	Robotic	Case Series	4	2015	5			(9–60)
Le Clerc	2015	France	Robotic	Case Series	4	2009–2013	12	7.3 (4.9–11)	61.9 (55–73)	31.2 (8–24)
Beyer	2016	Germany	RRP	Case Series	4	1992–2013	20	18.7	64.5^a^	24.7^a^
Iizuka	2016	Japan	Robotic	Case Series	4	2011–2015	3	12.1 (8.6–17)	61.6 (59–66)	18.3 (8–24)
Moreno Sierra	2016	Spain	Robotic	Case Series	4	2012–2013	4	7.1 (4.3–9.9)	61.25 (54–68)	Not recorded
Pettenati	2016	France	Open Robotic LRP	Retrospective comparative	4	2000–2013	16	7.01 (4.36–19.9)	61 (51–72)	46.6
Plagakis	2016	Australia	Robotic	Case Report	4	2016	1	13	60	120
Tugcu	2017	Turkey	Robotic	Case Report	4	2018	1	3.77	71	
Wang	2017	Singapore	Robotic	Case Report	4	2017	1		61	12
Fang	2018	China	Robotic	Case Report	4	2016	1	11.82	62	21
Iwamoto	2018	Japan	Open—1/13 LRP—3/13 Robotic—3/13	Retrospective comparative	3	2008–2017	13	8.79^a^	61^a^	27^a^
Zeng	2018	USA	Robotic	Case Report	4	2018	1	6.65	65	3
Mistretta	2019	Italy	Robotic	Case Series	4	2012–2016	9	5.6^a^	60^a^	42^a^
Bratt	2020	Sweden	Open, Robotic	Retrospective comparative	3	1998–2016	13	10.8	63	102
Felber	2020	France	Robotic	Retrospective comparative	3	2008–2017	39	6.8^a^	62^a^	47.9^a^
Leonard	2020	France	Robotic	Retrospective comparative	3	2008–2016	27	8.9 (4.4–19)	63.3 (43–73)	34.9 (0.5–85.5)
Minami	2020	Japan	Robotic	Case Report	4	2020	1	4.97	72	21
Kobari	2021	Japan	Robotic	Case Report	4	2021	1	9.23	65	12
Shahait	2021	USA	Robotic	Case Series	4	2014–2019	14	6.9^a^	60.2^a^	12
Sirisopana	2021	Thailand	Open	Case Series	4	2008–2020	1	25.66	67	129
Sirisopana	2021	Thailand	LRP	Case Series	4	2008–2020	1	10.84	64	47
Sirisopana	2021	Thailand	Robotic	Case Series	4	2008–2020	3	50.4 (9.63–130)	73 (66–79)	33.3 (6–63)
Marra	2022	UK, Italy, France, Germany, Turkey	Robotic	Case Series	4	2009–2019	41	6.5^a^	60^a^	42^a^

Study	Gleason score (GS)	Biochemical recurrence (%)	Positive margin (%)	OS %	CSS%	Graft complications (%)
Pre‐transplant						
Kocak	GS 3 + 3 = 1	Not recorded	0	100	100	0
Tillou	Not recorded	Not recorded	10.5	100	100	0
	GS 3 + 2 = 1 GS 3 + 3 = 10 GS 3 + 4 = 2 GS 4 + 3 = 1	0	14.3	100	100	0
	GS 3 + 2 = 1 GS 3 + 3 = 3 GS 3 + 4 = 1	Not recorded	0	100	100	0
Chahwan	Not recorded	4.3	10.9	100	100	2.2
	GS 3 + 2 = 2 GS 3 + 3 = 18 GS 3 + 4 = 5 GS 4 + 3 = 1	Not recorded	14.3	100	100	Not recorded
	GS 3 + 2 = 3 GS 3 + 3 = 9 GS 3 + 4 = 2 GS 4 + 3 = 1	Not recorded	6.7	100	100	Not recorded
	GS 3 + 3 = 2 GS 3 + 4 = 1	Not recorded	0	100	100	Not recorded
Post‐transplant						
Kinahan	Well differentiated = 1/3 Moderately differentiated = 2/3	0	Not recorded	100	100	0
Morton	Not recorded	0	Not recorded	100	100	0
Multanen	GS 5 = 1/1	0	Not recorded	100	100	0
Yiou	GS 3 + 4 = 1/1	0	0	100	100	0
Campagnari	Not recorded	0	Not recorded	100	100	Not recorded
Hafron	GS 6 = 5/7 GS 7 = 2/7	14.3	28.6	100	100	0
Shah	GS 6 = 1/1 *on biopsy	0	0	100	100	0
Thomas	GS 3 + 4 = 2/3 GS 3 + 3 = 1/3	0	0	66.6	100	0
Antonopoulos	GS 6 = 8/8	0	10	100	100	0
Jhaveri	GS 3 + 4 = 1/1	0	0	100	100	0
Kleinclauss	GS 6 = 18/20 GS 7 = 1/20 GS ≥ 8 = 1/20	10	10	100	100	15; 2 patients sustained ureteric injuries repaired intraoperatively 1 patient sustained graft failure due to pelvic haematoma needing nephrostomy, balloon dilatation, ureteropelvic reanastomosis due to stricture recurrence
Doerfler	GS 3 + 4 = 1/1	0	0	100	100	0
Maestro	GS 7 = 2/2	0	0	100	100	0
Robert	GS ≤ 6 = 7/9 GS 7 = 2/9	0	11.1	100	100	11.1; Graft loss 6 months postoperatively due to iliac vein thrombosis
Hoda	GS 6 = 11/16 GS 7 = 5/16	0	6.2	100	100	0
Saema	GS 6 = 1/1	0	0	100	100	0
Detti	GS 9 = 1/1	100	100	100	100	0
Smith	GS 6 = 3/3	0	33.3	100	100	0
Ghazi	GS 3 + 4 = 1/1	0	0	100	100	0
Polcari	GS 6 = 2/7 GS 7 = 4/7 GS 4 + 5 = 1/7	14.3	28.6	100	100	0
Wagener	GS 3 + 4 = 1/1	0	0	100	100	0
Heidenreich	GS 6 = 11/16 GS 3 + 4 = 3/16 GS 8 = 1/16 Unreported = 1/16	0	6.25	100	100	0
Heidenreich	GS 6 = 2/7 GS 3 + 4 = 3/7 GS 4 + 3 = 2/7	0	14.3	100	100	0
Jenjitranant	GS 4 + 3 = 1/1	0	100	100	100	0
Aboumohamed	GS 6 = 3/5 GS 7 = 2/5	0	0	100	100	0
Le Clerc	GS 6 = 5/11 GS 3 + 4 = 6/11	18.2	36.4	100	100	8.3; Acute transient renal failure requiring nephrostomy secondary to retropubic haematoma
Beyer	GS 6 = 5/20 GS 3 + 4 = 11/20 GS 4 + 3 = 3/20 GS ≥ 8 = 1/20	Not recorded	30	90	100	5; Ureteric injury
Iizuka	GS 3 + 4 = 2/3 GS 4 + 3 = 1/3	33.3	0	100	100	0
Moreno Sierra	GS 6 = 2/4 GS 3 + 4 = 2/4	25	50	100	100	0
Pettenati	GS 6 = 11/16 GS 7 = 4/16 GS 8 = 1/16	18.7	6.7	93.7	93.7	Not recorded
Plagakis	GS 3 + 4 = 1/1	0	0	100	100	0
Tugcu	GS 3 + 4 = 1/1	Not recorded	0	100	100	0
Wang	GS 5 + 4 = 1/1	100	Not recorded	100	100	100; ureteric stenosis requiring reconstruction
Fang	GS 3 + 3 = 1/1	0	Not recorded	100	100	0
Iwamoto	GS 7 = 12/13 GS ≥ 8 = 1/13	30.8	46	100	100	0
Zeng	GS 5 + 4 = 1/1	100	100	100	100	0
Mistretta	GS 3 + 3 = 4/9 GS 3 + 4 = 3/9 GS 4 + 3 = 2/9	22.2	22.2	77.8	100	0
Marra	GS 3 + 3 = 9/13 GS 3 + 4 = 3/13 GS 4 + 3 = 1/13	Not recorded	Not recorded	77	100	Not recorded
Felber	ISUP 1 = 14/39 ISUP 2 = 18/39 ISUP 3 = 4/39 ISUP 5 = 3/39	7.7	13.2	Not recorded	Not recorded	0
Leonard	ISUP 1 = 12/27 ISUP 2 = 8/27 ISUP 3 = 5/27 ISUP 5 = 2/27	7.4	44.4	88.9	100	7.4
Minami	GS 4 + 3 = 1/1	0	0	100	100	0
Kobari	GS 3 + 3 = 1/1	0	0	100	100	0
Shahait	GS 3 + 4 = 8/14 GS 4 + 3 = 4/14 GS 4 + 5 = 2/14	21.4	28.6	Not recorded	Not recorded	0
Sirisopana	GS 4 + 5 = 1/1	100	100	0	100	0
Sirisopana	GS 3 + 3 = 1/1	0	0	0	100	0
Sirisopana	GS 3 + 4 = 1/3 GS 4 + 3 = 1/3 GS 4 + 5 = 1/3	66.6	66.6	100	100	0
Marra	GS 3 + 3 = 9/41 GS 3 + 4 = 24/41 GS 4 + 3 = 4/41 GS 4 + 4 = 2/41 GS 4 + 5 = 2/41	10	17	95	100	0

^a^ Values expressed as median.

^b^ Gleason score on biopsy.

^c^ Side of PLND not specified.

Subgroup analysis by prostatectomy technique was conducted as shown in Table 3. The majority of open cases (60%) were conducted in Gleason 6 prostate cancer patients. The majority of robotic cases (57%) were conducted in Gleason 7 prostate cancer patients. Open techniques (open and perineal) were on average 1 h faster than minimally invasive techniques (laparoscopic and robotic). Length of stay was quicker, and there was less blood loss in minimally invasive techniques.

**TABLE 3:** Comparison of different radical prostatectomy approaches.

	Open (*n* = 94)	Perineal (*n* = 15)	Lap (*n* = 21)	Robotic (*n* = 190)
Studies	12	3	7	25
Mean PSA (ng/mL)	9.0 (87 pts)	6.0 (15 pts)	8.8 (18 pts)	10.2 (66 pts)
Mean age (years)	61.5 (70 pts)	62.7 (15 pts)	60.4 (18 pts)	63.3 (70 pts)
Mean f/up period (months)	30.6 (69 pts)	29.1 (15 pts)	18.3 (18 pts)	25.5 (78 pts)
Mean time from transplant to diagnosis (months)	83.7 (70 pts)	86.5 (7 pts)	99.9 (16 pts)	109.0 (79 pts)
T Stage	≤T2 = 69 T3 = 19 T4 = 1 Unreported = 5	≤T2 = 11 T3 = 4	T ≤ 2 = 16 T3 = 2 Unreported = 3	T ≤ 2 = 132 T3 = 41 Unreported = 17
Gleason score	Well differentiated = 1 Moderately differentiated = 2 GS ≤ 6 = 56 GS 7 = 25 GS 8 = 3 GS 9 = 2 Unreported = 5	GS ≤ 6 = 7 GS 7 = 8	GS ≤ 6 = 11 GS 7 = 7	GS ≤ 6 = 57 GS 7 = 108 GS 8 = 2 GS 9 = 12 GS 10 = 1 Unreported = 10
PLND	Unilateral = 20 Bilateral = 17 Unspecified = 7	0	Unilateral = 1	Unilateral = 50 Bilateral = 7
Mean operating time (min)	144.1 (64 pts)	123.4 (14 pts)	211.2 (18 pts)	208.2 (83 pts)
Mean blood loss (mL)	472.1 (64 pts)	506.5 (14 pts)	348.4 (18 pts)	385.9 (72 pts)
Mean LOS (days)	10.2 (56 pts)	5.8 (14 pts)	3.5 (9 pts)	4.3 (65 pts)
Complications	Clavien ≤2 = 27 Clavien 3 = 7	Clavien ≤2 = 3	Clavien ≤2 = 3 Clavien 3 = 2	Clavien ≤2 = 33 Clavien 3 = 6 Clavien 4 = 3
BCR (%)	5.7 (70 pts)	6.7 (15 pts)	9.5 (21 pts)	13.1 (186 pts)
Positive margin (%)	15.6 (82 pts)	20.0 (15 pts)	5.6 (18 pts)	24.7 (176 pts)
Graft complications (%)	4.5 (88 pts)	0	5.6 (18 pts)	2.2 (178 pts)

### 3.6 Surgery post‐transplant primary outcomes

**FIGURE 2:**
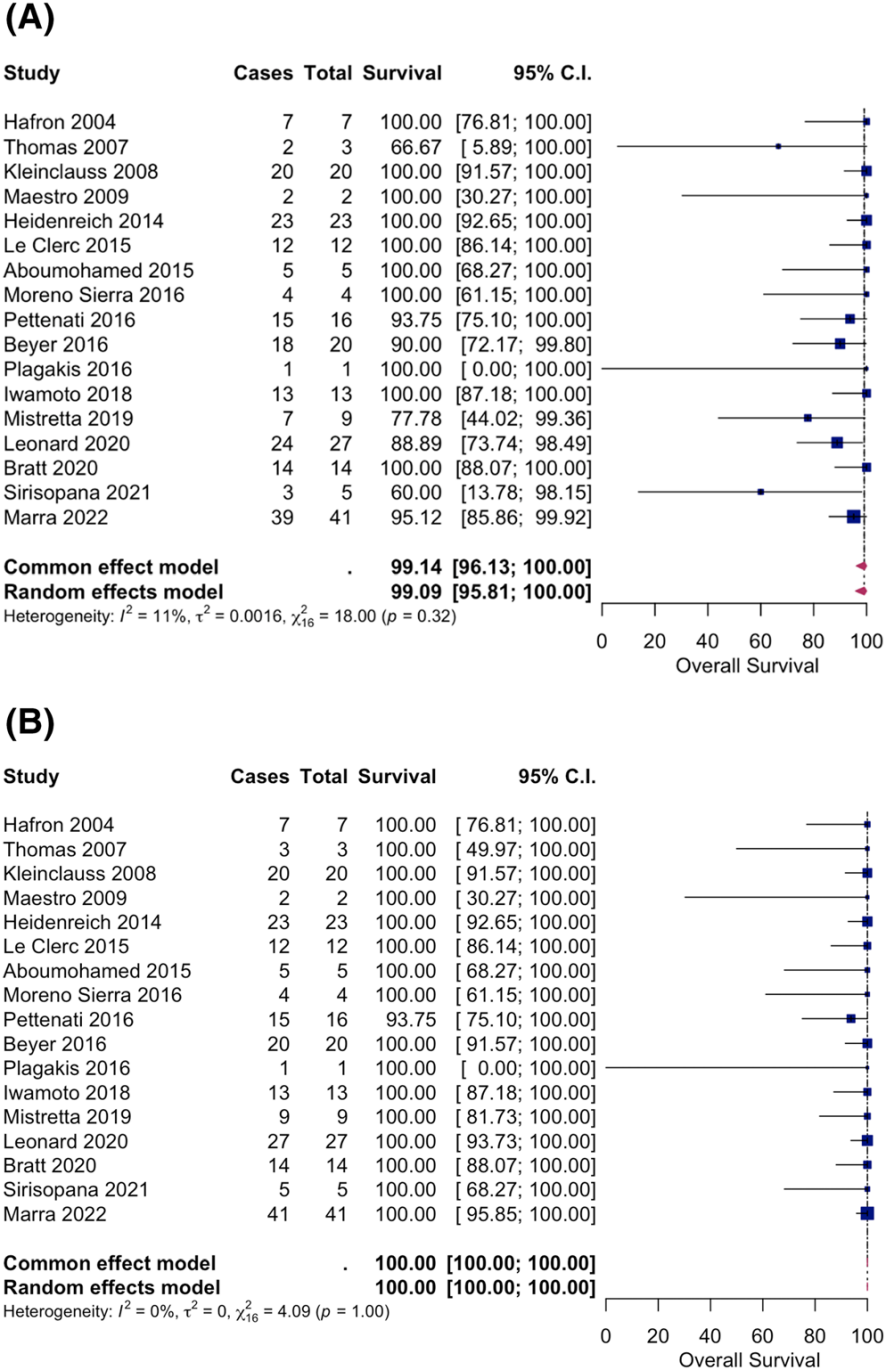
(A) Pooled analysis with random effects model—overall survival post‐transplant surgery 3 years. (B) Pooled analysis with random effects model—prostate cancer‐specific survival post‐transplant surgery 3 years.

Using a single‐arm random effects model, the overall survival rates at 1 and 3 years were 100% and 99%. The prostate cancer‐specific survival at 1 and 3 years was 100% (Figure 2A,B). There was one prostate cancer‐specific mortality that was in a Gleason 8 prostate cancer patient who eventually developed castrate‐resistant metastatic disease.^26^ There was a higher positive margin (24.7%) in the robotic group. Subgroup survival analysis by Gleason grading was unable to be performed because of the lack of clear reporting in the included studies.

### 3.7 Surgery post‐transplant secondary outcomes

The surgical complication rate was 14.5% with the vast majority of these being minor (Clavien Dindo Classification Grade 2 or less). Major complications included rectal injury and rectourethral fistula requiring colostomy,^17^ postoperative lymphocoele requiring percutaneous drainage or marsupialisation,^15^, ^27^, ^50^ postoperative bleeding requiring pelvic vessel angioembolisation^53^ and pelvic abscess requiring open drainage.^48^ The renal graft‐specific complication rate was 3% with the main cause being transplant ureteric injury during dissection.^15^, ^27^ Others included iliac vein thrombosis 6 months post‐surgery,^17^ pelvic haematoma resorting in renal graft hydronephrosis^70^ and ureteric stricture requiring subsequent reconstruction.^52^

Postoperative continence was reported in 58% of total surgical patients (192/330) with a pooled continence rate of 95%. Validated classification systems were not used. No data regarding continence rehabilitation schemes, degree of continence and time to full continence were reported. Postoperative erectile dysfunction was reported in 42% of total surgery patients with a pooled erectile dysfunction rate of 20%. No validated erectile dysfunction system or postoperative penile rehabilitation protocol was reported.

### 3.8 Surgery pre‐transplant

There were three studies that examined surgery treatment for prostate cancer before renal transplantation representing a total of 66 patients with a mean PSA of 8.2.^66^, ^67^, ^68^ The majority of patients had an open prostatectomy (64%). There were no studies examining robotic surgery in the pre‐transplant setting. Of the 66 patients, 54 (82%) had low‐risk prostate cancer (Gleason 6) and 12 (18%) patients had intermediate‐risk prostate cancer (Gleason 3 + 4). The mean time was 28.6 months between surgery treatment and renal transplantation.^69^ Mean operating time was 170 min. Length of stay, continence and erectile dysfunction rates were not recorded. These studies reported a 100% overall survival and 100% cancer‐specific survival at the last follow‐up.

### 3.9 Radiotherapy post‐transplant

Table 4 lists all radiotherapy patients with further supplementary data in Table A6. There was a total of 86 post‐transplant radiotherapy patients consisting of 54 external beam radiotherapy patients and 32 brachytherapy patients. The mean PSA was 11 ng/mL, and the mean patient age was 64 years. Gleason 6 patients contributed to 45% of cases, whereas Gleason 7 patients contributed to 42% of radiotherapy cases. Of the studies that listed the clinical staging, 73 patients (85%) had organ‐confined localised prostate cancer and six patients (7%) had cT3 disease. The range of doses for external beam radiotherapy was between 60 and 78 Gy. The dose for brachytherapy was 145 Gy. The mean time between renal transplant to radiotherapy was 94 months with a mean follow‐up period of 43 months. A total of 23 (26%) patients underwent either adjuvant or neoadjuvant androgen deprivation therapy (ADT) ranging from 6 months to 3 years duration. The criteria for undergoing adjuvant or neoadjuvant ADT was poorly reported with only one study listing the reason, for example cT3 disease.^26^

**TABLE 4:** Included radiotherapy studies.

Study	Year	Study country	Type of RT	Study type	Level of evidence	Accrual years	Patients	Mean PSA (range), ng/mL	Mean age (range), years	Mean follow‐up (range), months
Pre‐transplant										
Harada	2017	Japan	Brachytherapy	Case Report	4	2014	1	6.57	65	Not recorded
Chahwan	2017	France	EBRT	Case Series	4	2003–2015	4	6.7 (3.8–14)	65 (59–77)	25 (16–44)
Chahwan	2017	France	Brachytherapy	Case Series	4	2003–2015	2	6.9	59.9	Not recorded
Post‐transplant										
Mouzin	2004	France	RT	Case Series	4	1999–2003	8	15.4 (2.3–32.1)	65.1	28 (9–45)
Binsaleh	2011	Saudi Arabia	RT	Case Series	4	1980–2006	8	9.4 (1.4–3.1)	63.8 (58–77)	34.7 (2–98)
Beydoun	2014	Australia	Brachytherapy	Case Series	4	2002–2012	4	8.9	64* (61–66)	44 (12–60)
Rosenfelder	2014	UK	RT	Case Report	4	2014	1	Not recorded	60	48
Iizuka	2016	Japan	RT	Case Report	4	2012	1	5.1	70	43 (40–46)
Iizuka	2016	Japan	Brachytherapy	Case Report	4	2012	1	17.3	71	45 (38–52)
Pettanati	2016	France	Brachytherapy	Retrospective comparative	3	2000–2013	3	5.13 (4.3–6.38)	69.3 (68–71)	46.6
Pettanati	2016	France	RT	Retrospective comparative	3	2000–2013	4	12.63 (2.7–30)	70 (63–78)	
Rivero‐Belenchon	2018	Spain	Brachytherapy	Case Series	4	2000–2015	8	6.25 (±1.97)	64.4	48
Tasaki	2019	Japan	Brachytherapy	Case Series	4	2007–2018	3	7.6 (4.5–10.4)	64.7	42.7 (34–50)
Gojdic	2019	Slovakia	Brachytherapy	Case Series	4	2003–2016	3	10.24 (8.94–12.24)	57.8 (56–59.6)	49 (30–73)
Gojdic	2019	Slovakia	RT	Case Series	4	2003–2016	1	7.82	62.8	
Bratt	2020	Sweden	Brachytherapy	Retrospective comparative	3	2000–2016	9	17.68	63.6	74.66
Bratt	2020	Sweden	RT	Retrospective comparative	3	2000–2016	22	19	64.3	59.4
Ileana	2020	Mexico	RT	Case Series	4	2013–2018	2	9.6 (4.8–14.4)	60	40 (20–60)
Detti	2021	France	RT	Case Series	4	1998–2017	6	7.36 (4.7–16)	60.8	59.2

Study	Gleason score	Radiation dose, Gy	OS %	CSS%	Graft complications
Pre‐transplant					
Harada	GS 3 + 4 = 1/1	Not recorded	100	100	Not recorded
Chahwan	GS 3 + 3 = 2/4 GS 3 + 4 = 2/4	Not recorded	100	100	Not recorded
Chahwan	GS 3 + 2 = 1/2 GS 3 + 4 = 1/2	Not recorded	100	100	Not recorded
Post‐transplant					
Mouzin	bGS ≤ 6 = 6/8 bGS 7 = 2/8	70	75	100	0
Binsaleh	bGS 6 = 3/8 bGS 7 = 4/8 bGS 8 = 1/8	60–66	87.5	87.5	0
Beydoun	bGS 7 = 3/4 bGS 8 = 1/4	Not recorded	100	100	0
Rosenfelder	bGS 4 + 3 = 1/1	74	100	100	0
Iizuka	bGS 6 = 1/2 bGS 7 = 1/2	74	100	100	0
Iizuka	bGS 6 = 1/2 bGS 9 = 1/2	144	100	100	0
Pettanati	bGS 6 = 3/3	145	100	100	0
Pettanati	bGS 7 = 2/4 bGS 8 = 1/4 bGS 9 = 1/4	74	75	75	0
Rivero‐Belenchon	bGS 3 + 3 = 8/8	145	62.5	100	0
Tasaki	bGS 3 + 3 = 1/3 bGS 3 + 4 = 1/3 bGS 4 + 4 = 1/3	39–145	100	100	0
Gojdic	bGS 3 + 3 = 3/3	145	100	100	0
Gojdic	bGS 4 + 3 = 1/1	74	100	100	0
Bratt	GS 3 + 3 = 4/9 GS 3 + 4 = 3/9 GS 4 + 3 = 1/9 GS 8 = 1/9	Not recorded	78	100	Not recorded
Bratt	GS 3 + 3 = 4/22 GS 3 + 4 = 7/22 GS 4 + 3 = 8/22 GS 8 = 1/22 GS 9 = 1/22	Not recorded	77	91	Not recorded
Ileana	bGS 3 + 3 = 1/2 pGS 4 + 4 = 1/2	66 to 78	100	100	0
Detti	bGS 6 = 2/6 bGS 3 + 4 = 1/6 bGS 4 + 5 = 1/6 Unreported = 2/6	70 to 76	83.3	100	0

### 3.10 Radiation primary outcome (oncological)

**FIGURE 3:**
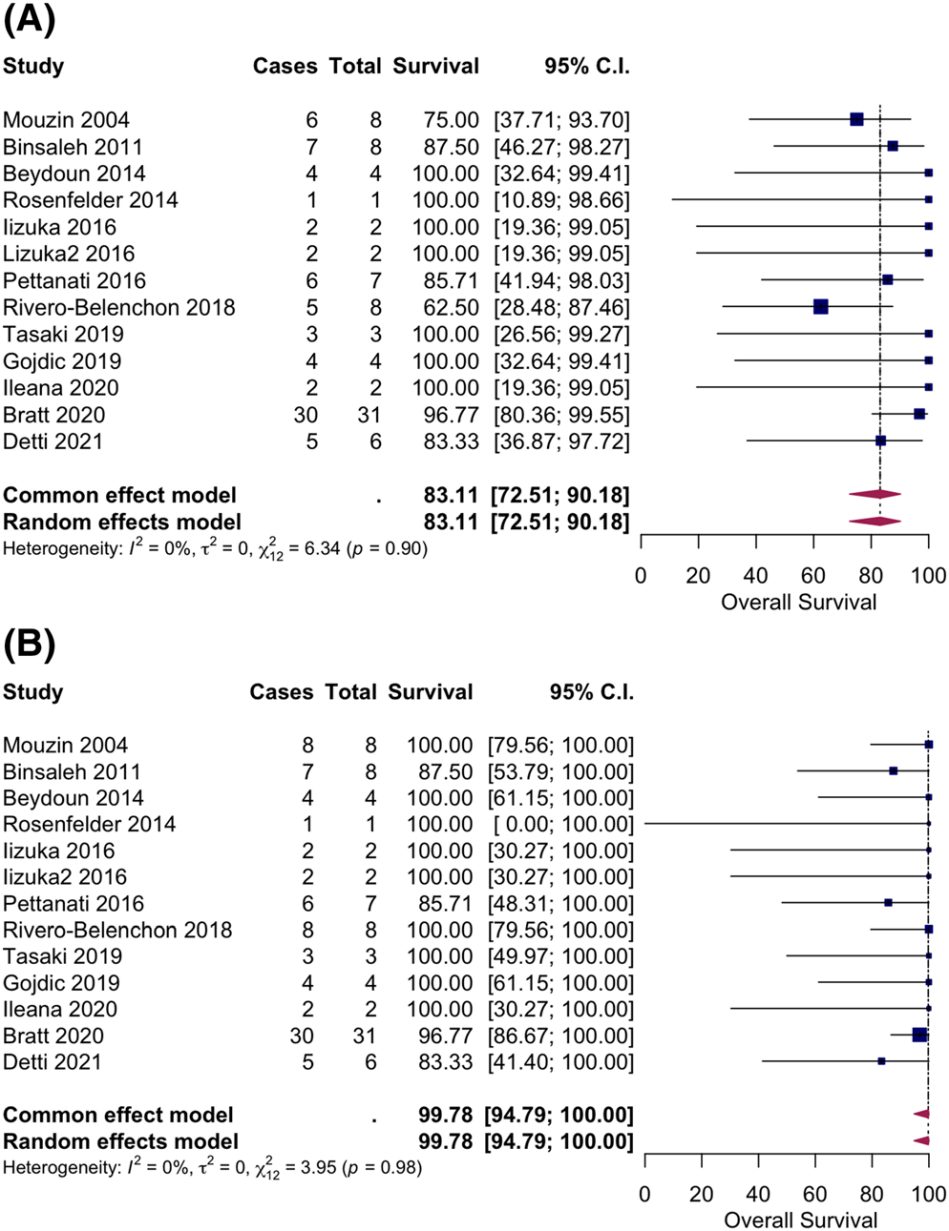
(A) Pooled analysis with random effects model—overall survival post‐transplant radiation therapy 3 years. (B) Pooled analysis with random effects model—prostate cancer‐specific survival post‐transplant radiation therapy 3 years.

The overall survival rate at 1 and 3 years was 100% and 83%. The prostate cancer‐specific survival at 1 and 3 years was 100% and 99% (Figure 3A,B). The prostate cancer deaths were in high‐risk Gleason 8 prostate cancer patients.^3^, ^26^, ^54^, ^60^ Subgroup analysis by Gleason grading again was unable to be performed because of the lack of clear reporting in the included studies.

### 3.11 Radiation secondary outcome (functional)

Only half of the radiation patients included had complications specifically stated. The majority of complications were minor grade 1 cystitis and proctitis. There was one major complication—one patient who had a grade 3 proctitis requiring a diverting colostomy.^60^

### 3.12 Radiotherapy pre‐transplant

Overall, there were two studies that examined radiotherapy treatment for prostate cancer before renal transplantation.^67^, ^69^ There were a total of seven patients with a mean age of 63.5 and PSA of 6.7. Of the seven patients, three had low‐risk prostate cancer (Gleason 6) and four patients had intermediate‐risk prostate cancer (Gleason 3 + 4). Only one study listed the mean time (19 months) between radiotherapy treatment cessation and renal transplantation.^69^ These studies reported a 100% overall survival and 100% cancer‐specific survival at the last follow‐up. Radiation complications were not recorded.

### 3.13 Active surveillance

There were two studies that included active surveillance with a total of 21 cases.^3^, ^26^ The surveillance protocol was not stated. There was no prostate cancer‐specific death recorded. Five out of the 21 cases (25%) died of non‐prostate cancer causes specifically myocardial infarction, chronic kidney disease, leukaemia and renal abscess. Two patients had Gleason 4 + 3, which does not fit into contemporary active surveillance guidelines. The surveillance follow‐up duration was between 1 and 9 years for included patients.

### 3.14 Focal therapy

There was only one focal therapy study included.^65^ This case report examined a 62‐year‐old male with a PSA 6 and Gleason 7 prostate cancer who underwent high‐intensity frequency ultrasound (HIFU) treatment. The patient was still alive with no recurrence at a follow‐up of 42 months. No complications were recorded.

### 3.15 Surgical comparison with non‐renal transplant cohorts with prostate cancer

In total, there were eight retrospective comparative studies. Of these, six studies compared oncological outcomes of radical prostatectomy in RTRs with standard non‐RTR prostate cancer control groups.^15^, ^17^, ^19^, ^26^, ^47^, ^50^ One study compared open retropubic radical prostatectomy versus open perineal radical prostatectomy amongst RTRs.^22^ One study compared prostate cancer outcomes in renal transplant patients with non‐transplant patients however did not report subgroup analysis based on treatment type, for example surgery and radiotherapy.^3^

Amongst the studies comparing the oncological outcomes of radical prostatectomy in RTRs with standard non‐RTR control groups, the surgical margin, biochemical recurrence, cancer‐specific survival and overall survival rates were similar in both the cases and control. The blood loss, hospital length of stay and surgery duration were similar in all comparative studies. Functional outcomes were poorly reported with only three studies reporting continence with no difference between the transplant and control groups.^15^, ^17^, ^47^

### 3.16 Immunosuppression regimes

Immunosuppression regimes were poorly reported across all studies with some studies reporting medication uptake by percentage of study cohort, whereas other studies reported according to specific combination therapy. Steroids were the most commonly reported immunosuppressant followed by calcineurin inhibitors. A comparison between calcineurin inhibitors versus mTOR inhibitors was unable to be conducted because of variations in immunosuppression drug reporting across all studies.

### 3.17 Heterogeneity assessment

There was a low risk of heterogeneity with all survival outcomes being <20% on the I‐squared test.

### 3.18 Certainty of evidence

Recommendations for clinical practice were graded by the modified GRADE methodology.^10^ The rating strength was low based on the poor overall quality of the evidence and the certainty of the results from retrospective studies.

**Table:** 

Recommendation	Certainty rating
Surgery and radiotherapy have equal oncological efficacy in localised prostate cancer treatment in post‐renal transplant patients. There is growing data on active surveillance and focal therapy.	Low
The timing of localised prostate cancer treatment in renal transplant patients should be determined by risk stratification, e.g. grading and use of nomogram.	Low
List patients for renal transplantation with a diagnosis of low and favourable intermediate‐risk prostate cancer without additional delay.	Low
Unfavourable intermediate‐risk (Gleason 4 + 3) and high‐risk prostate cancer should be treated prior to renal transplantation.	Low
Prostate cancer treatment in renal transplant patients should be conducted in a dedicated tertiary‐level transplant hospital.	Low

## 4 DISCUSSION

### 4.1 Key findings

The majority of prostate cancer patients were treated post‐renal transplant. The majority of prostate cancer cases were low‐ to intermediate‐risk prostate cancer with a mean follow‐up of 3 years. With regard to the primary outcome, there was high overall and cancer‐specific survival amongst the RTRs who underwent prostate cancer surgery and radiotherapy comparable with the general non‐transplant population. These findings are in keeping with survival rates in other reviews in this field.^71^, ^72^

At 3 years, the cancer‐specific survival was similar between both groups (99% in radiation group and 100% in surgery group). The overall survival was higher in the surgery group (99% vs. 83%). Although patient comorbidities were not collected because of poor reporting amongst included studies, this may be related to radiation patients being generally less fit and older. Despite data showing equivalence between surgery and radiation for localised prostate cancer, this selection bias is well known in contemporary prostate cancer management with surgery preferred for younger, less co‐morbid patients.^73^ This bias may also explain the reason why the majority of studies published had patients treated with surgery (86%). Any future research must ensure a higher proportion of non‐surgical treatment options.

All prostate cancer‐specific mortality cases were in high‐risk prostate cancer patients. High‐risk prostate cancer should be treated regardless if it is found pre‐ or post‐renal transplant. This finding highlights the importance of a risk‐based model, such as the American Society of Transplantation recommended wait time guideline,^74^ to guide prostate cancer management in the renal transplant patient.

Graft complications were higher in the surgery group although the lack of complications reported in the radiation group may be due to the short follow‐up period. The main graft complication was graft ureteric injury in four patients. Transplant ureteric stenting placed prior to prostatectomy may aid in ureter identification preventing injury, especially during the endopelvic fascia dissection and PLND.^75^ There was one graft loss recorded that was a case of late deep vein thrombosis affecting the iliac vessels leading to graft loss.^17^ This case highlights the importance of prophylactic postoperative DVT prophylaxis. Although that patient did not undergo a PLND, this case also highlights the importance of selective PLND based on nomograms or radiological pelvic lymph node involvement considering the higher risk of DVT with PLND.^76^

Prostate cancer treatment complications were similar in the radiation and surgery groups. Serious complications (Clavien Dindo Grade 3 or higher) occurred in 6% of surgery patients with no perioperative deaths recorded. The radiation complications were mainly related to cystitis and proctitis with the most severe case being a patient with Gleason 6 prostate cancer who underwent EBRT and developed colitis requiring a diverting colostomy.^60^

### 4.2 Implications of surgical approach in renal transplant patients

The kidney graft location within the iliac fossa provides unique anatomical challenges and technical modifications including laparoscopic port placement and dissection. Suggested modifications for RARP include placement of all ports on the opposite side to the graft, emphasis on placing ports under direct vision because of previous abdominal surgery and initiation of bladder mobilisation and space of retzius development from the contralateral side to the graft.^21^ Other modifications include using one instead of two assistant ports and not using the fourth robotic arm.^24^, ^25^ For open radical prostatectomy, Heidenreich recommended the blade of the self‐retained to be placed above the rectus muscle to avoid pressure injury to the graft.^22^

The vast majority of robotic cases were conducted in the standard manner with only seven patients across three studies reporting the retzius sparing approach.^34^, ^44^, ^46^ There was no difference between the standard and retzius sparing approaches. The shortened length of stay and lower blood loss associated with robotic surgery in this review is in keeping with the reported literature.^77^ The finding of interest however is the high positive margin rate in prostatectomy cases in RTRs (21%) with the highest rate in the robotic arm (25%). Contributing factors may include the fact that the robotic arm had a higher proportion of intermediate‐risk prostate cancer (57%) compared with the open arm, which had a higher proportion of indolent Gleason 6 low‐risk disease (60%). In addition to the modifications listed above, a smaller pelvic space and altered tissue planes from prior renal transplant can lead to a more challenging dissection. Careful meticulous dissection combined with an experienced surgical team and a general or transplant surgeon on standby is helpful to prevent the previously listed complications.

The vast majority of studies did not list the indication for a PLND. When it was performed, the vast majority were conducted on the contralateral side only. This is not particularly surprising considering the risks of an ipsilateral lymph node dissection including transplant ureteric and vascular injury resulting in graft loss. Unless there is preoperative imaging suggestive of ipsilateral pelvic lymph node metastases, it is recommended that dissection is avoided on that side.

### 4.3 Implications of radiotherapy approach in renal transplant patients

The location of the renal graft within the iliac fossa impacts radiotherapy planning considering its close proximity to the prostate. Kidneys are radiosensitive with radiation nephritis and ureteric stricture potential long‐term risks especially if the ipsilateral iliac lymph nodes are part of the treatment field. The pathogenesis is progressive microvascular injury and stromal fibrosis leading to relative ischaemia and stricture formation.^59^ Measures to decrease the complication risk revolve around decreasing the dose to surrounding organs such as irradiation when the bladder is full or decreasing the planning target volume specifically to avoid the upper pelvic areas.^36^

### 4.4 Lack of active surveillance and focal therapy papers

Despite the widespread contemporary use of active surveillance for low‐grade prostate cancer in non‐transplant patients, there were only two studies that described its use.^3^, ^26^ The majority of included studies were in the pre‐active surveillance era. As such, a large proportion of included patients with low‐grade disease (Gleason 6) had treatment.

Active surveillance in renal transplant patients appears to be safe. A large Swedish registry found that immunosuppression in renal transplant patients did not increase the risk of prostate cancer progression.^3^ They also found that transplant recipients were not more likely than age‐matched non‐transplant men to be diagnosed with any high‐risk or metastatic prostate cancer. This suggests that active surveillance is an appropriate option in immunosuppressed patients.

### 4.5 Prostate cancer screening

The reporting of PSA screening for prostate cancer renal transplant patients was limited. In a survey of US transplant centres, 89% routinely screen for prostate cancer in renal transplant candidates and patients with the most common starting age being 50 years old.^78^ The concern of routine pre‐transplant screening is the impact of overscreening and overtreatment. This review found that half of treated localised prostate cancer patients had low‐grade Gleason 6 disease with only a small portion (21 patients) placed on active surveillance. This would indicate a significant degree of overtreatment for these patients.

The practice of subjecting men with low‐grade prostate cancer to treatment and prolonged subsequent follow‐up before they can be accepted for an organ transplantation can therefore be questioned, not least as longer time on dialysis is associated with worse outcomes after kidney transplantation.^79^ It is suggested that the decision to transplant or not should take into account the comparison between prostate cancer mortality versus dialysis mortality. The overall 5‐year survival for renal dialysis varies between 35.8% and 50% dependent on age and comorbidities.^80^ Renal transplantation has shown to significantly improve overall mortality by up to 86% at 5 years.^80^ It is noted that the vast majority of localised prostate cancers have 5‐year cancer‐specific survival rates in excess of 90%.^81^

### 4.6 Limitations of research

Overall included studies were limited to retrospective comparative and case series. Most studies had a serious to critical risk of bias during analysis. The majority of studies were from one centre with participants entering at different stages of disease with short follow‐up to detect any clinically relevant oncological outcome. There was significant heterogeneity with regard to inclusion criteria, outcome measures and prostate cancer‐specific mortality definitions amongst studies. Most studies did not report outcome measures a priori. In particular, functional outcomes including quality of life measures for continence and erectile function were not measured with standardised tools.

### 4.7 Limitation of review processes

All Gleason 7 intermediate‐risk cases were grouped together as the majority of studies did not separately report ISUP Grade Group 2 to Grade Group 3. Based on population‐based active surveillance data, it would be inferred that certain favourable intermediate‐risk prostate cancer would be amenable to active surveillance; however, no strong conclusion can be made with this data set.^82^ Any future papers in this field must clearly differentiate the different grade subgroups. In addition, the survival time points included were short considering the decades‐long duration required to assess long‐term prostate cancer survival.^73^ The 3‐year survival data presented were directly related to the lack of long‐term follow‐up in the included studies.

### 4.8 Implications for future research

As there are relatively low numbers of renal transplant patients who have prostate cancer, recruitment for an adequately powered future RCT may be problematic. Bratt reported a prevalence of 0.07% of prostate cancer patients with a prior renal transplant in Sweden.^3^ Practically, a well‐designed prospectively collated registry study crosslinking renal transplant and prostate cancer data would be the ideal study type.

Future research needs to be multicentred encompassing multiple national and international renal transplant centres with subgroup analysis by treatment type. There should be more emphasis on recruiting patients undergoing active surveillance, radiotherapy and focal therapy. Future reporting of functional and complication outcomes such as continence would benefit from standardised measures such as the validated Incontinence Questionnaire‐Urinary Incontinence Short Form.^83^ Any future research should also examine the impact of different immunosuppression regimes specifically a comparison between mTOR inhibitors (with reported anti‐neoplastic properties) versus calcineurin inhibitors (with reported pro‐neoplastic properties). From an oncological‐specific perspective, the grading and staging should be uniform with long‐term follow‐up (10 years or more).

### 4.9 Implications of results for practice and policy

Prostate cancer management in RTRs should be conducted in tertiary renal transplant centres with specialised uro‐oncology expertise. The results indicate that future studies require a uniform prostate cancer screening program for renal transplant candidates and recipients to be reported as part of their trial. Although there are numerous criteria available, a commonly accepted one from the American Society of Transplantation would involve biennial PSA screening from the age of 50 with a life expectancy of 10 years or more before and after renal transplantation.^84^ The results of this systematic review suggest that the majority of localised prostate cancer diagnosed in RTRs are low grade. Active surveillance should be the primary management option in this group considering the financial and public health medical implications of overtreatment.

## 5 CONCLUSIONS

Localised prostate cancer treatment in renal transplant patients should be risk stratified according to cancer risk nomograms. Surgery or radiation treatment for localised prostate cancer in renal transplant patients appears equally efficacious. Given the limitations of this study, there is a trend that low and favourable intermediate‐risk prostate cancer patients may proceed to renal transplantation without cancer treatment. High‐risk prostate cancer should be treated prior to renal transplantation if detected pre‐transplant.

## AUTHOR CONTRIBUTIONS

Anthony Dat, Gavin Wei, Simon Knight and Weranja Ranasinghe contributed to the design and implementation of the research, to the analysis of the results and to the writing of the manuscript.

## CONFLICT OF INTEREST STATEMENT

None of the authors have a conflict of interest to disclose.

## APPENDIX A APPENDICES

**TABLE A1:** Search string.

Patients	
Prostate cancer renal transplant	
1	exp KIDNEY TRANSPLANTATION/
2	(kidney$1 or renal$) adj5 (transplant$ or graft$ or allograft$).ti,ab.
3	exp PROSTATIC NEOPLASMS/
4	(prostat* adj5 (cancer or neopl*)).ti,ab.
5	1 OR 2
6	3 OR 4
7	5 AND 6

Intervention	
Active surveillance	
8	exp WATCHFUL WAITING/
9	active surveill*.ti,ab.
10	OR/8–9

Control	
Surgery	
11	exp PROSTATECTOMY/
12	prostatectomy.ti,ab.
13	OR/11‐12
Radiotherapy	
14	exp RADIOTHERAPY/
15	radiotherapy.ti,ab.
16	OR/14‐15
Brachytherapy	
17	exp BRACHYTHERAPY/
18	brachytherapy.ti,ab.
19	OR/17‐18

Combined	
20	7 AND (10 OR 13 OR 16 OR 19)

**TABLE A2:** Excluded studies.

Paper	Year	Country	Reason
Hevia	2018	Italy UK	Review
Marra	2018	Italy	Review
Bieri	2020	USA	Simulation Study
Kim	2017	Korea	Different population: end stage renal failure without proceeding to renal transplant
Boissier	2013	France	Different population: prostate cancer in non‐transplant population
Keenan	2021	Ireland	Different population: heart and lung transplant
Chiurchiu	2010	Argentina	Different intervention: everolimus
Okamura	2009	Japan	Different population: donor
Drachenberg	2003	USA	Different population: RCC
Giessing	2015	Germany	Different population: all urological follow‐up post‐renal transplant
Sherer	2017	USA	Review
Martini	2020	Italy	Review on RARP technique
Stein	1984	USA	Different population: melanoma
Gin	2016	USA	Survey
Manson	1989	Canada	Letter
Gunther	1978	Germany	Different population: renal stones
Sihra	2019	UK	Different population: post‐prostatectomy lymphocoele presenting with renal failure
Abe	2005	Japan	Different population: bladder cancer
Brendler	1999	USA	Letter
Chabchoub	2005	France	Letter
Kane	2013	USA	Letter
Lopez Martin	2011	Spain	Letter
Lawrence	2015	USA	Different population: TURP xenograft
Ball	2016	USA	Letter
Wetterauer	2015	Germany	Different population: lymphoma in prostate cancer xenograft
Ozcelik	2015	Turkey	Review
Floyd	2015	UK	Different population: TCC Bladder
Tillou	2014	France	Review
Rodriguez Faba	2015	Spain	Review
Iannetti	2014	Italy	Different population: RCC
Sun	2003	China	Different population: lymphocoele
Iselin	1994	UK	Different population: lymphocoele
Schonberger	2002	Germany	Review
Wenzel	2021	Germany	Different population: kidney, bone, heart, lung, liver transplant
Aminsharifi	2021	USA	Review
Beyer	2009	USA	Review prostate cancer screening
Tanaka	2009	Japan	Review laparoscopic surgery guidelines
Soloway	2008	USA	Different population: recurrence prostate cancer
Al Ekish	2013	USA	Met initial population criteria however no outcomes reported (Cryotherapy in renal transplant patients)
Kreydin	2013	USA	Different population: non‐transplant population
Heldt	2011	USA	Different population: patient population in ESRF patients however no subsequent renal transplantation
Sforza	2019	Italy	Different population: nodal recurrence post RRP in post‐renal transplant pt
Thompson	2008	USA	Different population: renal, liver and heart transplant—No breakdown by organ.
Coombs	2012	USA	Different population: brachytherapy for prostate cancer heart and renal transplant—no breakdown by organ.
Konety	1998	USA	Different population: liver, heart and renal with no breakdown by organ, biopsy grade poorly defined (well, moderate and poor) not used in contemporary practice.
Waeckel	2021	France	Different population—liver, cardiac and heart with no breakdown by organ or treatment group.
Hata	2018	Japan	Survey donor candidates
Zilinska	2017	Slovakia	Different population: multiple cancers after renal transplant
Kleinclauss	2016	France	Review
Doerfler	2008	France	Duplicate of included study in different language (French)
Secin	2004	USA	Different population: pre‐transplant prostatectomy however no subsequent renal transplant
Lechevallier	2002	France	Review
Cormier	2003	France	Prostate Cancer in Renal Transplant patients—outcome measures unable to be separated by treatment type and prostate cancer grading
Keinclauss	2008	France	Prostate Cancer in Renal Transplant patients—outcome measures unable to be separated by treatment type and prostate cancer grading
Elkentaoui	2010	France	Prostate Cancer in Renal Transplant patients—outcome measures unable to be separated by treatment type and prostate cancer grading
Melchior	2011	Germany	Prostate Cancer in Renal Transplant patients—outcome measures unable to be separated by treatment type and prostate cancer grading
Karczewiski	2012	Poland	Prostate Cancer in Renal Transplant patients—outcome measures unable to be separated by treatment type and prostate cancer grading
Hevia	2014	Spain	Prostate Cancer in Renal Transplant patients—outcome measures unable to be separated by treatment type and prostate cancer grading
Carvalho	2017	Portugal	Prostate Cancer in Renal Transplant patients—outcome measures unable to be separated by treatment type and prostate cancer grading
Narvaez	2018	Spain	Prostate Cancer in Renal Transplant patients—outcome measures unable to be separated by treatment type and prostate cancer grading
Haroon	2019	Ireland	Prostate Cancer in Renal Transplant patients—outcome measures unable to be separated by treatment type and prostate cancer grading
Spatafora	2021	Italy	Prostate Cancer in Renal Transplant patients—outcome measures unable to be separated by treatment type and prostate cancer grading

**TABLE A3:** Risk of bias table retrospective comparative studies.

Study	Year	Study country	Bias due to confounding	Bias in selection of participants into study	Bias in classifications of interventions	Bias due to deviations from intended interventions	Bias due to missing data	Bias in measurement of outcomes	Bias in selection of reported result	Overall bias
Keinclauss	2008	France	Moderate	Critical	Serious	Low	Low	Serious	Low	Critical
Robert	2009	France	Serious	Critical	Serious	Low	Low	Serious	Moderate	Critical
Hoda	2010	Germany	Critical	Critical	Serious	Low	Low	Serious	Low	Critical
Heidenreich	2014	Germany	Moderate	Critical	Serious	Low	Low	Serious	Low	Critical
Leonard	2020	France	Moderate	Critical	Serious	Low	Low	Serious	Low	Critical
Felber	2020	France	Serious	Critical	Serious	Low	Low	Serious	Moderate	Critical
Pettanati	2016	France	Serious	Critical	Serious	Low	Low	Serious	Low	Critical
Bratt	2020	Sweden	Moderate	Low	Moderate	Low	Low	Moderate	Low	Moderate

**TABLE A4:** Risk of bias table case series and report.

Study	Year	Study country	Aim of study cleared stated	Study conducted prospectively	Participant characteristics described	Cases collected >1 centre	Eligibility criteria clearly stated	Participants recruited consecutively	Did participants enter study at similar point of disease?	Intervention clearly described?	Additional interventions described?
Kinahan	1991	Canada	Partial	No	Partial	No	No	Unclear	Yes	Yes	No
Morton	1995	USA	Partial	No	No	No	No	Unclear	No	Yes	No
Campagnari	2002	Brazil	Partial	No	Yes	No	No	Unclear	Yes	Yes	No
Hafron	2004	USA	Partial	No	Yes	No	Yes	Yes	No	Yes	No
Antonopoulos	2008	Brazil	Partial	No	Yes	No	Yes	Yes	Yes	Yes	No
Maestro	2009	Spain	Yes	No	Yes	No	Yes	Unclear	Yes	Yes	Yes
Smith	2011	USA	Yes	No	Yes	No	Yes	Unclear	Yes	Yes	No
Polcari	2012	USA	Yes	No	Yes	No	Yes	Unclear	No	Yes	No
Le Clerc	2015	France	Yes	No	Yes	No	Partial	Unclear	Yes	Yes	No
Iizuka	2016	Japan	Partial	No	Yes	No	Yes	Unclear	No	Yes	No
Moreno Sierra	2016	Spain	Yes	No	Yes	No	Yes	Unclear	No	Yes	No
Beyer	2016	Germany	Yes	No	Yes	No	Yes	Unclear	No	Yes	No
Yiou	1999	France	Partial	No	Partial	No	Partial	Unclear	Yes	Yes	No
Shah	2006	USA	Yes	No	Yes	No	Yes	Yes	Yes	Yes	No
Jhaveri	2008	USA	Yes	No	Yes	No	Yes	Yes	Yes	Yes	No
Doerfler	2009	France	Yes	No	Yes	No	Yes	Yes	Yes	Yes	No
Wagener	2012	Germany	Yes	No	Yes	No	Yes	Yes	Yes	Yes	No
Saema	2010	Thailand	Yes	No	Yes	No	Yes	Yes	Yes	Yes	No
Jenjitranant	2014	Thailand	Yes	No	Yes	No	Yes	Yes	Yes	Yes	No
Ghazi	2012	USA	Yes	No	Yes	No	Yes	Yes	Yes	Yes	Yes
Detti	2011	Italy	Yes	No	Yes	No	Yes	Yes	Yes	Yes	No
Multanen	1998	Finland	Yes	No	Yes	No	Yes	Yes	Yes	Yes	No
Zeng	2018	USA	Yes	No	Yes	No	Yes	Yes	Yes	Yes	No
Plagakis	2016	Australia	Yes	No	Yes	No	Yes	Yes	Yes	Yes	No
Minami	2020	Japan	Yes	No	Yes	No	Yes	Yes	Yes	Yes	No
Tugcu	2017	Turkey	Yes	No	Yes	No	Yes	Yes	Yes	Yes	No
Thomas	2007	USA	Yes	No	Yes	No	Yes	Unclear	No	Yes	No
Sirisopana	2021	Thailand	Yes	No	Yes	No	Yes	Unclear	No	Yes	No
Mistretta	2019	Italy	Yes	No	Yes	No	Yes	Unclear	No	Yes	No
Shahait	2021	USA	Yes	No	Yes	No	Yes	Unclear	No	Yes	No
Kobari	2021	Japan	Yes	No	Yes	No	Yes	Yes	Yes	Yes	No
Iwamoto	2018	Japan	Yes	No	Yes	No	Yes	Unclear	No	Yes	No
Fang	2018	China	Yes	No	Yes	No	Yes	Yes	Yes	Yes	No
Aboumohamed	2015	USA	Yes	No	No	No	No	Unclear	Unclear	Yes	No
Wang	2017	Singapore	Yes	No	Yes	No	Yes	Yes	Yes	Yes	No
Marra	2022	UK, France, Turkey, Germany, Italy	Yes	No	Yes	Yes	Yes	Unclear	No	Yes	Yes
Binsaleh	2011	Saudi Arabia	Partial	No	Yes	Yes	No	Unclear	No	Yes	Yes
Beydoun	2014	Australia	Yes	No	Yes	No	Yes	Unclear	Yes	Yes	No
Iizuka	2016	Japan	Partial	No	Yes	No	Yes	Unclear	No	Yes	No
Iizuka	2016	Japan	Partial	No	Yes	No	Yes	Unclear	No	Yes	No
Rosenfelder	2014	UK	Yes	No	Yes	No	Yes	Yes	Yes	Yes	No
Mouzin	2004	France	Yes	No	Yes	No	Yes	Unclear	No	Yes	No
Detti	2021	France	Yes	No	Yes	No	Yes	Unclear	No	Yes	No
Ileana	2020	Mexico	Yes	No	Yes	No	Yes	Unclear	No	Yes	No
Tasaki	2019	Japan	Yes	No	Yes	No	Yes	Unclear	No	Yes	Yes
Rivero‐Belenchon	2018	Spain	Yes	No	Yes	No	Yes	Unclear	Yes	Yes	Yes
Gojdic	2019	Slovakia	Yes	No	Yes	No	Yes	Unclear	Yes	Yes	Yes
Narvaez	2018	Spain	Yes	No	Yes	No	Yes	Yes	No	Yes	Yes
Carvalho	2017	Portugal	Yes	No	Yes	No	Yes	Yes	No	Yes	Yes
Melchior	2011	Germany	Unclear	No	Partial	No	Partial	Unclear	Yes	Yes	Yes
Cormier	2003	France	Yes	No	No	Yes	No	Unclear	No	Partial	Yes
Elkentaoui	2010	France	Unclear	No	No	No	Yes	Yes	No	Yes	Yes
Spatafora	2021	Italy	Yes	No	Yes	No	Yes	Unclear	No	Yes	Yes
Karczewiski	2012	Poland	Unclear	No	Yes	No	Partial	Unclear	No	Yes	Yes
Hevia	2014	Spain	Yes	No	Partial	No	No	Unclear	No	Yes	Yes
Lledo	2005	Spain	Yes	No	Yes	No	No	No	Yes	Yes	Yes
Kocak	2009	Turkey	Yes	No	Yes	No	Yes	Unclear	Yes	Yes	No
Chahwan	2017	France	Yes	No	Yes	Yes	Yes	Unclear	No	Yes	Yes
Tillou	2014	France	Yes	No	Yes	Yes	Yes	Unclear	No	Yes	Yes
Harada	2017	Japan	Yes	No	Yes	No	Yes	Yes	Yes	Yes	No

Study	Outcome measures described a priori	Were outcome assessors blinded to the intervention that patients received?	Outcomes measured with appropriate objective or subjective measures	Outcomes measured before and after intervention	Statistical measures used to measure outcome appropriately	Length of follow‐up reported and long enough for important events and outcomes to occur (>5 years)	Loss of follow‐up reported	Does the study provide estimates of the random variability in the data analysis of relevant outcomes?	Adverse events reported	Conclusions supported by results	Are both competing interests and sources of support for the study reported?
Kinahan	Partial	No	Partial	No	Unclear	No	No	No	Partial	Yes	No
Morton	No	No	Partial	No	Yes	No	Yes	No	No	Yes	No
Campagnari	Yes	No	Partial	No	Yes	Yes	Yes	No	Yes	Yes	No
Hafron	Yes	No	Partial	No	Yes	No	Yes	No	Yes	Yes	Yes
Antonopoulos	No	No	Partial	No	Unclear	No	Unclear	Yes	Partial	Unclear	No
Maestro	Partial	No	Partial	No	Yes	No	Yes	No	Yes	Yes	Yes
Smith	No	No	Partial	No	Yes	Yes	Yes	No	Yes	Yes	Yes
Polcari	Yes	No	Partial	No	Yes	Yes	Yes	No	Yes	Unclear	No
Le Clerc	Yes	No	Partial	No	Yes	No	Yes	No	Yes	Yes	No
Iizuka	Partial	No	Partial	No	Unclear	No	Yes	No	Partial	Yes	No
Moreno Sierra	Partial	No	Partial	No	Yes	No	Yes	No	Partial	Unclear	Yes
Beyer	Yes	No	Partial	No	Yes	No	Yes	No	Partial	Unclear	Yes
Yiou	Partial	No	Partial	No	Unclear	No	Yes	No	Partial	Yes	No
Shah	Yes	No	Partial	No	Yes	No	Yes	No	Partial	Yes	No
Jhaveri	Yes	No	Partial	No	Yes	No	Yes	No	Partial	Unclear	Yes
Doerfler	Yes	No	Partial	No	Yes	No	Yes	No	Partial	Unclear	No
Wagener	Yes	No	Partial	No	Yes	No	Yes	No	Partial	Yes	No
Saema	No	No	Partial	No	Yes	No	Yes	No	Yes	Yes	Yes
Jenjitranant	Yes	No	Partial	No	Yes	No	Yes	No	Yes	Yes	No
Ghazi	Yes	No	Partial	No	Yes	No	Yes	No	Partial	Unclear	No
Detti	Yes	No	Partial	No	Yes	No	Yes	No	Yes	Yes	Yes
Multanen	Yes	No	Partial	No	Yes	No	Yes	No	Yes	Yes	No
Zeng	No	No	Partial	No	Yes	No	Yes	No	Yes	Yes	Yes
Plagakis	Yes	No	Partial	No	Yes	No	Yes	No	Partial	Yes	Yes
Minami	Yes	No	Partial	No	Yes	No	Yes	No	Yes	Unclear	Yes
Tugcu	Yes	No	Partial	No	Yes	No	Yes	No	Partial	Yes	Yes
Thomas	Yes	No	Partial	No	Yes	No	Yes	No	Yes	Yes	No
Sirisopana	Yes	No	Partial	No	Yes	No	Yes	No	Yes	Yes	Yes
Mistretta	Yes	No	Partial	No	Yes	No	Yes	No	Yes	Yes	Yes
Shahait	Yes	No	Partial	No	Yes	No	Yes	No	No	Unclear	No
Kobari	Yes	No	Partial	No	Yes	No	Yes	No	Partial	Yes	No
Iwamoto	Yes	No	Partial	No	Yes	No	Yes	No	Yes	Yes	No
Fang	Yes	No	Partial	No	Yes	No	Yes	No	Partial	Yes	No
Aboumohamed	No	No	Partial	No	Yes	No	Yes	No	Partial	Unclear	No
Wang	Yes	No	Partial	No	Yes	No	Yes	No	Yes	Yes	No
Marra	Yes	No	Partial	No	Yes	No	Yes	Yes	Yes	Yes	Yes
Binsaleh	No	No	Partial	No	Unclear	No	Yes	No	No	Unclear	No
Beydoun	Yes	No	Partial	No	Yes	No	Yes	Yes	Yes	Yes	Yes
Iizuka	No	No	Partial	No	Yes	No	Yes	No	Yes	Unclear	Yes
Iizuka	No	No	Partial	No	Yes	No	Yes	Yes	Yes	Yes	No
Rosenfelder	Partial	No	Partial	No	Yes	No	Yes	No	Yes	Unclear	No
Mouzin	Partial	No	Partial	No	Yes	No	Yes	No	Yes	Yes	No
Detti	Yes	No	Partial	No	Yes	No	Yes	No	Yes	Yes	Yes
Ileana	Yes	No	Partial	No	Yes	No	Yes	No	Yes	Unclear	Yes
Tasaki	Yes	No	Partial	No	Yes	No	Yes	No	Yes	Yes	Yes
Rivero‐Belenchon	Yes	No	Partial	No	Yes	No	Yes	Yes	Yes	Yes	No
Gojdic	Yes	No	Partial	No	Yes	No	Yes	No	Yes	Unclear	No
Narvaez	Yes	No	Partial	No	Yes	Yes	Yes	No	Partial	Yes	Yes
Carvalho	No	No	Partial	No	Yes	Yes	Yes	Yes	Partial	Unclear	No
Melchior	No	No	Partial	No	Unclear	Yes	Yes	No	No	Yes	No
Cormier	No	No	Partial	No	Yes	Yes	Yes	No	Yes	Yes	No
Elkentaoui	No	No	Partial	No	Unclear	No	No	No	No	Unclear	No
Spatafora	Yes	No	Partial	No	Yes	Yes	Yes	Yes	Partial	Yes	Yes
Karczewiski	Yes	No	Partial	No	Yes	No	No	No	No	Unclear	No
Hevia	No	No	Partial	No	Yes	Yes	Yes	No	Yes	Yes	No
Lledo	Yes	No	Partial	No	Yes	Yes	Yes	Yes	Partial	Unclear	Yes
Kocak	Yes	No	Unclear	No	Yes	No	Yes	No	Partial	Yes	Yes
Chahwan	Yes	No	Unclear	No	Unclear	No	No	Yes	Partial	Unclear	Yes
Tillou	Yes	No	Unclear	No	Unclear	No	No	Yes	Partial	Unclear	Yes
Harada	Yes	No	Unclear	No	Yes	No	Yes	No	Partial	Yes	No

**TABLE A5:** Further included surgery studies characteristics.

Study	Mean time from transplant to diagnosis (range), months	Immunotherapy Scheme	pT stage	PLND	Mean operating time (range), minutes	Mean blood loss (range), mL	Mean length of stay (range), days	Complications (Surgery)	Continence (%)	Erectile dysfunction (%)
Pre‐transplant										
Kocak	NA	Tacrolimus, MMF, Pred	Not recorded	Not recorded	Not recorded	Not recorded	Not recorded	Not recorded	Not recorded	Not recorded
Tillou	NA	Tacrolimus, MMF, Pred	Not recorded	Bilateral = 8	Not recorded	Not recorded	Not recorded	Not recorded	Not recorded	Not recorded
			T2a = 2 T2b = 4 T2c = 6 T3a = 3	Not recorded				Clavien 1 = 6 Clavien 2 = 1 Clavien 3b = 2		
			T2c = 4 T3a = 1	Not recorded				Not recorded		
Chahwan	NA	Tacrolimus, MMF, Pred	Not recorded	Not recorded	Not recorded	Not recorded	Not recorded	Not recorded	Not recorded	Not recorded
			T2a = 8 T2b = 5 T2c = 12 T3a = 3	Unspecified = 13				Clavien 1 = 3 Clavien 2 = 1 Clavien 3 = 11 Clavien 4 = 1		
			T2a: 2 T2b = 2 T2c = 7 T3a = 3	Unspecified = 3				Clavien 1 = 2 Clavien 2 = 1 Clavien 3 = 3		
			T2c = 2 T3a = 1	Not recorded				Clavien 3 = 1		
Post‐transplant										
Kinahan	72	AZA + CNI + steroids	A1 = 1/3 A2 = 2/3	Unilateral = 1/1	133 (120–150)	1466 (1200–2000)	10 (8–12)	Clavien 2: 3/3 transfusion 1/3 UTI treated with antibiotics	66	33
Morton	114 (60–168)	AZA + steroids = 2, CNI + steroids = 1	Not recorded	Not performed	Not recorded	Not recorded	Not recorded	Not recorded	Not recorded	Not recorded
Multanen	48	MMG, AZA, CNI	Not recorded	Unilateral = 1/1	Not recorded	Not recorded	Not recorded	Not recorded	100	100
Yiou	Not recorded	Not recorded	T2 = 1/1	Not performed	Not recorded	Not recorded	Not recorded	Not recorded	100	Not recorded
Campagnari	56.5 (41–72)	AZA + steroids	T1c = 1/2 T2a = 1/2	Not performed	Not recorded	Not recorded	Not recorded	Not recorded	Not recorded	Not recorded
Hafron	86.5 (24–192)	CNI (tacro) + steroids = 3, CNI (cyclosporin) + steroids = 3, MMF + steroids + mTOR = 1	T2a = 2/7 T2b = 2/7 T2c = 3/7	Not performed	92.7 (83–115)	492.9 (100–1500)	2.6 (2–5)	Clavien 2: 1/7 transfusion	Not recorded	Not recorded
Shah	Not recorded	MMF, CNI, Steroids	T2a = 1/1	Not performed	215	200	2	0	100	0
Thomas	62 (18–144)	1. Sirolimus, MMF, steroids 2. Sirolimus, Tacrolimus, steroids 3. None	T2c = 3/3	Not performed	236.7 (180–290)	425 (75–1000)	3.3 (2–5)	Clavien 1: 1/3 small urine leak Clavien 2: 1/3 transfusion	Not recorded	Not recorded
Antonopoulos	89.9 (40–209)	CNI 50%, CNI + AZA 35%, CNI + MMF 15%	T2a = 1/8 T2c = 7/8	Unilateral = 8/8	183 (150–240)	656 (100–2000)		Clavien 2: 2/8 transfusion	75	Not recorded
Jhaveri	Not recorded	Not recorded	T2c = 1/1	Bilateral = 1/1	200	400	7	Clavien 2: 1/1 transfusion	100	0
Kleinclauss	72	CNI (40%), CNI + AZA (35%), CNI + MMF (15%)	T2a = 5/20 T2b = 10/20 T3a = 3/20 T3b = 2/20	Unilateral = 10/10	163	516	11.9	Clavien 1: 2/20 haematoma 1/20 urinary leakage requiring IDC 1/20 haematuria requiring IDC Clavien 2: 3/20 UTI 3/20 urosepsis 2/20 transfusion Clavien 3: 1/20 graft failure due to pelvic haematoma needing nephrostomy, balloon dilatation, ureteropelvic reanastomosis due to stricture recurrence 1/20 lymphocoele drained percutaneously 2/20 ureteric injuries repaired intraoperatively	65	75
Doerfler	Not recorded	MMF + CNI + Steroids	T2c = 1/1	Not performed	130	50	3	0	100	Not recorded
Maestro	144 (108–180)	CNI, MMR, steroid *n* = 1	T2a = 1/1 T2c = 1/1	Not performed	200 (180–220)	300 (200–400)	3.5 (3–4)	0	100	0
Robert	101 (16–219)	Not recorded	T2 = 8/9 T3 *n* = 1/9	Unilateral = 1/1	214	394		Clavien 3: 2/9 rectal injuries repaired intraoperatively; one patient developed rectal fistula requiring colostomy and repair.	Not recorded	Not recorded
Hoda	81.2 (28–219)	CNI + steroids + MMF *n* = 16	T2a = 2/16 T2b = 4/16 T2c = 10/16	Not performed	108.3 (88–188)	211.1 (128–498)	10.1 (7–18)	Clavien 1: 1/16 urine leak requiring prolonged IDC Clavien 2: 1/16 transfusion	Not recorded	Not recorded
Saema	108	NA	T3a = 1/1	Not performed	210	300	4	0	100	Not recorded
Detti	120	Sirolimus, MMF	T3b = 1/1	Bilateral = 1/1	Not recorded	Not recorded	Not recorded	Not recorded	Not recorded	Not recorded
Smith	Not recorded	Not recorded	T2c = 3/3	Not performed	322 (244–400)	75 (50–100)	2.3 (2–3)	0	100	Not recorded
Ghazi	480	Not recorded	Not recorded	Not performed	130	125	Not recorded	0	Not recorded	Not recorded
Polcari	99.6 (6–155)	CNI + steroids + AZA/MMF *n* = 6, CNI + MMF *n* = 1	T2c = 3/7 T3a = 4/7	Unilateral 4/4	186 (110–240)	Not recorded	1.8 (1–3)	Clavien 2: 1/7 transfusion 1/7 atrial fibrillation 1/7 urosepsis	Not recorded	Not recorded
Wagener	324	CNI	T2c = 1/1	Unilateral 1/1	220	300	Not recorded	0	100	0
Heidenreich	95 (24–206)	Not recorded	T2 = 13/16 T3 = 3/16	Bilateral 16/16	125 (105–215)	390 (100–1500)	7.9 (5–13)	Clavien 1: 1/16 wound infection	Not recorded	40
Heidenreich		Not recorded	T2 = 3/7 T3 = 4/7	Not performed	154 (132–215)	520 (250–1500)	9 (5–14)	Clavien 1: 2/7 wound infection	Not recorded	
Jenjitranant	252	MMF + CNI	T2c = 1/1	Not performed	210	250	6	0	100	0
Aboumohamed	Not recorded	Not recorded	T2 = 4/5 T3a = 1/5	Not performed	Not recorded	Not recorded	Not recorded	0	100	Not recorded
Le Clerc	79.7 (17–242)	CNI (91.7%), AZA (83.3%), steroids (41.7%)	T2 = 9/11 T3a = 2/11	Not performed	241	646.8 (10–1200)	Not recorded	Clavien 2: 1/12 transfusion Clavien 3: 1/12 Retropubic haematoma with subsequent acute transient renal failure requiring nephrostomy + clot drainage Other: 1/12 Unable to perform prostatectomy despite conversion to open; subsequently treated with radiotherapy	Not recorded	Not recorded
Beyer	Not recorded	Not recorded	T2a = 2/20 T2c = 8/20 T3a = 7/20 T3b = 2/20 T4 = 1/20	7^+^	180*	550*	Not recorded	Clavien 1: 2/20 prolonged IDC Clavien 2: 4/20 transfusion 1/20 urosepsis Clavien 3: 1/20 ureteric injury 1/20 lymphocoele 1/20 haematoma	Not recorded	Not recorded
Iizuka	156 (84–240)	CNI + AZA + steroids *n* = 1, Tac + MMF + steroids *n* = 1, FK + MMF + steroid + everolimus *n* = 1	T2 = 3/3	Not performed	161 (127–195)	51.6 (30–75)	8 (7–9)	Clavien 2: 1/3 difficulty in urination	Not recorded	Not recorded
Moreno Sierra	120	Not recorded	Not recorded	Not recorded	196	Not recorded	Not recorded	0	100	0
Pettenati	117 (15–402)	Not recorded	T2a = 4/16 T2c = 10/16 T3a = 2/16	Not performed	Not recorded	Not recorded	Not recorded	Not recorded	Not recorded	Not recorded
Plagakis	Not recorded	Not recorded	T2c = 1/1	Not performed	139	190	2	0	100	0
Tugcu	4	Not recorded	Not recorded	Not performed	110	60	3	0	Not recorded	Not recorded
Wang	72	Not recorded	T3b = 1/1	Unilateral = 1/1	Not recorded	Not recorded	Not recorded	Clavien 3b: 1/1 ureteric stenosis requiring reconstruction	Not recorded	Not recorded
Fang	144	Tacrolimus, MMF, steroids	T2c = 1/1	Unilateral = 1/1	230	200	13	0	100	Not recorded
Iwamoto	136*	Tacrolimus, MMF, steroids ×9, Cyclosporine A, MMF, steroids ×2, Cyclosporine A, azathioprine, steroids ×2	T2 = 10/13 T3 = 3/13	Not performed	158*	42*	6.5*	Clavien 3b: 1/13 pelvic abscess secondary to urine leak requiring open drainage	Not recorded	Not recorded
Zeng	5	Not recorded	T3b = 1/1	Unilateral = 1/1	207	500	3	Clavien 2: 1/1 transfusion	Not recorded	Not recorded
Mistretta	108*	Not recorded	T2a = 1/9 T2c = 6/9 T3a = 1/9 T3b = 1/9	Unilateral = 1/2 Bilateral = 1/2	160*	100*	4*	Clavien 2: 1/9 UTI	77.8	44.4
Bratt	146	Not recorded	T1c = 9 T2 = 3 Tx = 1	Not recorded	Not recorded	Not recorded	Not recorded	Not recorded	Not recorded	Not recorded
Felber	58*	Not recorded	T2a 5/39 T2b 2/39 T2c 21/39 T3a 9/39 T3b 2/39	Unilateral = 12/13 Bilateral = 1/13	180*	150*	4*	Clavien ≤2: 16/39 Clavien 3b: 2/39 ‐ lymphocoele treated by lap marsupialisation Clavien 4a: 1/39 ICU admission for bradycardia 1/39 ICU admission for electrolyte disorders	68.6	87.1
Leonard	101.6 (17.5–270.2)	Not recorded	T2a = 15/27 T2b = 9/27 T2c = 2/27 T3a = 1/27	Unilateral = 5/7 Bilateral = 2/7	244 (120–480)	571.3 (100–1500)	5.7 (3–16)	Clavien 2: 6/27 Clavien 3b: 1/27 haematoma of Retzius space with ureteral compression Clavien 4a: 1/27 haematoma of Retzius space with ureteral compression leading to AKI and APO	96	Not recorded
Minami	96	Not recorded	T2a = 1/1	Not performed	208	50	7	0	100	Not recorded
Kobari	24	Not recorded	T2a = 1/1	Not performed	187	100	8	0	100	Not recorded
Shahait	97.2	Not recorded	T2 = 8/14 T3 = 6/14	Unilateral = 14/14	129.7	110	1	0	87.5	Not recorded
Sirisopana	156	Not recorded	T3b = 1/1	Not performed	365	630	13	Clavien 2: 1/1 transfusion	100	Not recorded
Sirisopana	108	Not recorded	T2a = 1/1	Not performed	210	300	6	Clavien 2: 1/1 transfusion	100	Not recorded
Sirisopana	168 (96–252)	Not recorded	T2a = 1/3 T3b = 1/3 T2c = 1/3	Not performed	203.3 (190–210)	166.67 (100–250)	6.7 (5–8)	0	100	Not recorded
Marra	118*	mTOR 1 (2.4%) Antimetabolites 2 (4.9%) CNI 31 (75.6%) Steroids 12 (29.2%)	T2 = 29/41 T3 = 11/41 Unreported = 1/41	Unilateral = 10/12 Bilateral = 2/12	201*	300*	4*	Clavien 2: 2/41 UTI 1/7 AKI secondary to glomerulonephritis Clavien 3a: 1/7 Haemorrhage requiring embolization	86	18.2

**TABLE A6:** Further included radiotherapy studies characteristics.

Study	Mean time from transplant to diagnosis (months)	Immunotherapy scheme	Clinical T stage	ADT (%)	Complications (Radiation) CT CAE	Prostate cancer recurrence %
Pre‐transplant						
Harada	NA	Tacrolimus, MMF, Prednisolone	T1c = 1/1	Not recorded	Not recorded	0
Chahwan	NA	Tacrolimus, MMF, Prednisolone	T1b = 1/4 T1c = 2/4 T2 = 1/4	Not recorded	Not recorded	0
Chahwan	NA	Tacrolimus, MMF, Prednisolone	T1c = 1/2 T2 = 1/2	Not recorded	Not recorded	0
Post‐transplant						
Mouzin	Not recorded	CNI + AZA + steroids	T1c = 2/8 T2a = 4/8 T2b = 1/8 T3a = 1/8	87.5	G1 diarrhoea = 5/8 Rectal irritation and urgency with mucus in stools = 2/8 G1 cystitis = 4/8 G2 cystitis = 1/8	25
Binsaleh	80.25 (18–276)	CNI, AZA, steroids *n* = 4 CNI, MMF, steroids *n* = 3 CNI, AZA, steroids *n* = 1 CNI, mMF, steroids *n* = 1	T1b = 1/8 T1c = 4/8 T2 = 3/8	50	Not recorded	0
Beydoun	13 (6–17)	Cyclosporine *n* = 2 Tacrolimus *n* = 1 Sirolimus *n* = 1 MMF *n* = 1 Prednisolone *n* = 3	T1c = 3/4 T2a = 1/4	0	Late G1 = 1/4 (voiding symptoms managed with alpha blockers)	0
Rosenfelder	156	MMF + CNI + Steroids	T3bN1 = 1/4	100	Not recorded	0
Iizuka	126 (84–168)	MMF + CNI + Steroids = 1	T1c = 1/2T2a = 1/2	Not recorded	G1 cystitis = 1/2	0
Iizuka	180 (4–26)	MMF + CNI + Steroids = 1	T1c = 2/2	Not recorded	G1 cystitis = 2/2	0
Pettanati	53.7 (12–134)	Not recorded	T1c = 1/3 T2a = 2/3	Not recorded	Not recorded	0
Pettanati	10.5 (2–21)		T2b = 1/4 T3a = 2/4 T3b = 1/4	75	Not recorded	25
Rivero‐Belenchon	79.2	Cyclosporine A, MMF, steroids *n* = 1 Tacrolimus, MMF, Steroids *n* = 4 Everolimus, MMF, steroids *n* = 1 Tacrolimus + MMF *n* = 1 Deflazacort + tacrolimus *n* = 1	T1c *n* = 7/8 T2a *n* = 1/8	0	Not recorded	0
Tasaki	88 (48–120)	Cyclosporine A, MMF, Steroids *n* = 2 Tacrolimus, MMF, Steroids *n* = 1	T1c = 3/3	33.3	G1 cystitis = 3/3	0
Gojdic	50.7 (6–108)	Sirolimus, steroids *n* = 1 Tacrolimus, MMF, *n* = 1 Tacrolimus, MMF, steroids *n* = 1	T1c = 1/3 T2c = 2/3	100	Late GU: G2 UUI = 1/4 Late GU: G2 Urethral stricture = 1/4	0
Gojdic	1	Tacrolimus, MMF, steroids *n* = 1	T2c = 1/1	100	Not recorded	0
Bratt	147.6	Not recorded	T1c = 7/9 T2 = 2/9	Not recorded	Not recorded	Not recorded
Bratt	171.8	Not recorded	T1c = 11/22 T2 = 9/22 T3 = 1/22 TX = 1/22	Not recorded	Not recorded	Not recorded
Ileana	275 (68–482)	Tacrolimus, MMF, Steroids; *n* = 1 AZA + steroids *n* = 1	cT2a = 1/2 pT2 = 1/2	50	Late GU: G2 Bladder neck contracture = 1/2	0
Detti	91.9 (48–144)	Steroids + other immunosuppressive agents = 1 Sirolimus, MMF, prednisolone *n* = 1 Tacrolimus, everolimus, corticosteroids = 1	Not recorded	16.66666667	Acute GU: G1 cystitis = 4/6 Acute GI: G1 proctitis = 2/6 G2 proctitis = 1/6 Late GI: G2 proctitis = 1/6 G3 proctitis requiring colostomy = 1/6	0
